# Supplementary material for: “Tell them you smoke, you’ll get more breaks”: a qualitative study of occupational and social contexts of young adult smoking in Scotland
Source: BMJ Open. 2018 Dec 31;8(12):e023951. doi: 10.1136/bmjopen-2018-023951 (PMC6318505; doi:10.1136/bmjopen-2018-023951)
Supplement: Supplementary file 1 [file bmjopen-2018-023951supp001.pdf]

Interview Guide

The interviews aim to explore:

- views of smoking, current smoking behaviour and history
- influence of peers/family/home/social networks on smoking beliefs and behaviours
- influence of social contexts – the home, local area, educational and occupational environments (and transitions)
- mechanisms for stopping smoking; what has helped smoking cessation; use of and views of NRT/e-cigarettes/vaping devices
- views, awareness and use of e-cigarettes
- (views of smoking policy/legislation)
- **For smokers**
- barriers and facilitators to quitting, and intention to quit in the future
- barriers/facilitators to interventions/support to help quit
- intention to quit in the future
- perceptions of the impact of their smoking on their own health/lives
- **For Ex-smokers**
- why and how they stopped smoking
- what helped smoking cessation: support and facilitating factors
- perceptions of any specific interventions/support they have experienced
- **For never smoked**:
- factors/issues that may be protective in preventing smoking initiation
- influence of peers and social groups, local community and social context

<u>Introduction/Recap</u>

To explore perceptions and experiences of smoking and quitting including views of smoking, current smoking behaviour/ history and influence of peers/family/home/social networks and context on smoking beliefs and behaviours. Study funded by Cancer Research UK.

Interview Format

- Format of depth interview (open questions, hearing their views)
- Use of grids to help completion of interview
- No right or wrong answers – their views are important/ Confidentiality
- Withdrawal at any time from interview as whole, or in not answering particular questions
- Timing of interview

Recording of Interview

- Digital recording of interviews – check they are happy with this
- Report, use of quotations, anonymization/ Check if respondent has any questions? happy to proceed?

Consent

- Obtain informed consent.

Introduction/ context (5 mins)

The aim here is to create a picture of the participant and their social context. The types of questions you could ask to get started are – Tell me a bit about yourself?

Check age and remind participant that they have been contacted as they previously took part in SHeS 2014 or 2015

Who do you live with, if anyone? Relationship with others in household/ Describe the community you live in, what is it like, how long have you lived there?

Work status and other sampling characteristics (10mins)

The aim of this section is to understand their current educational/employment status. The kind of questions to ask in this section are:

We are interested in what has been happening in your life since you were recently/around 16 years old. Can you tell us about what has been going on for you?

- Are you currently in school/college/university; employed or unemployed? **USE TIMELINE AND SHOW TO RESPONDENT TO USE AS A PROMPT.**

  Map out individual's school, work, university, training and periods of unemployment using **timeline grid (Column A)**. The following probes could be used:

  - If school/further education: what stage? What next?
  - Employed: Who with? How long for? Why change jobs? Do you like the job?
  - Unemployed? How long for? Are you receiving benefits? Any interviews, training? Any voluntary work? Youth projects?
  - Future hopes and plans

  Then map out individual's social context/life changes **(Column B)**. The following probes could be used:

  - Where were you living? Who with? Why the changes?
  - Were you renting? Own the property/pay mortgage? Parents/others own?
  - Life events: any major changes? Becoming a parent?
  - Future hopes and plans

  - ONLY IF THE INTERVIEWEES RAISE THESE ISSUES THEMSELVES PLEASE PROBE: Significant deaths of family members/ friends? Serious illnesses?
  - Problems with police, law. Note any times in young offender institutions, prison, fines, CPOs etc

Then identify **smoking status**:

- Have you ever tried smoking tobacco?
- *If never tried, go to* **<u>Never smoked section</u>**

Smoking initiation (5 mins)

We are interested in your smoking history from when you first tried it to today; let's start with when you first tried it…. (Please use ***Timeline Column C*** *for this smoking history section) Use questions such as:*

- Can you tell me all the reasons you tried smoking? (Probe fully on this) When was this? What age? Who with? How did you start? What did you think when you first tried it? Did you feel any pressure to smoke at all and who from? Did you grow up in a household where anyone smoked, and if so who did?

- What did you smoke when you first tried smoking? Tobacco, Roll-ups, Cannabis etc / who did you smoke with? Did you carry on smoking? Where did you smoke, when and how many - weekdays vs weekends? In what ways did you obtain tobacco (direct purchase, agent purchase, from friends, etc)? Which shops/friends/family members, etc?

- **If spontaneously mentions e-cigs record in Timeline Column D and probe fully.**

History for smokers – Current and ever smokers (15- 25 mins)

This section is for those who smoke or ever smoked and we are interested in gathering a full detailed history of behaviour and exposure to smoking as well as current status. Use ***Timeline Column C*** *for history*

Of particular interest are experiences and any changes during and after school, from initiation through the years 16 to current age. **Particularly interested in transitions** *e.g. school to work.*

Ask in detail about ***main changes*** *in smoking over course of history to current day -and* ***any factors associated with these changes****.* Here it will be important to probe on what factors cause an increase/decrease in smoking (e.g. drinking/socialising with friends; feeling anxious/stressed/unhappy; access to money; feelings of stigma, transitions etc)
Questions such as: So thinking of when you first started smoking, please take me through your smoking history. We are interested in any changes that have occurred over time.

From when you first started smoking to nowadays:

- What smoked? Type, Brand, how many – weekdays, weekends, during day vs evenings, etc – probe on reasons behind any reported changes. Cannabis smoking?

- Where usually smoke?*, ask about : different locations/nights out; home (any smoking rules?) etc*

- When did you smoke and who with? (day/ weekends /evenings)? What else were they doing when they were smoking? *Probe for smoking and drinking relationship* friends and family smoking?

- Why did you smoke? What did you enjoy and not enjoy about smoking? Any particular triggers?

- Any periods of cutting down or smoking more frequently? Why was this?

Quitting for Current smokers only (5-10 mins)

Quit Attempts: If attempted to quit at any time *then go through first and most recent attempt in detail (if multiple attempts) to understand process using* ***Column C (****and* ***Column D if e-cigs used)****: what prompted decision to quit, interventions aware of at the time that prompted or helped them start, get through it, what didn't help, what was offered, not offered, why went back to smoking etc. For both first and last attempt, ask questions such as (can use* ***Timeline*** *to help):*

- Why did you try to stop smoking then? How long did this attempt last? Did you manage to stop or reduce your smoking? Why did you start again?:
    - What helped/hindered the attempt? Prompts: were you aware of any leaflets, websites, ads, information? If yes, how did these help or not? School or college-based education or info?
    - Offered smoking any cessation support? Tools, aids such as nicotine gum, nicotine patches, electronic cigarette, vapes etc? Prescription, purchased or obtained in other ways? How did these help, or not?
- What is the longest period of time you have ever managed to stop smoking? What made you start again on this occasion?
- Do you want to give up smoking *now*? What, if anything, encourages you to think about quitting? What holds you back from quitting?
- Do you think you will always smoke? What if anything would make you think about stopping in the future?

Ex-Smoker 15-20 mins

Ask about first quitting attempt, if multiple, and then the successful attempt using **Timeline** ***Column C (****and* ***Column D*** *if e-cigs used****).*** *Probe fully on process for first and successful time, from decision to quit to starting again and anything that was different to previous attempts, and why successful attempt was different*

- When did you first try to quit? What made you <u>consider</u> quitting at this stage? Take me through this quit attempt - how did you go about it?:
    - What helped/hindered during this quit attempt? Prompts: were you aware of any leaflets, websites, ads, information? If yes, how did these help or not? School or college-based education or info?
    - Offered smoking any cessation support? Tools, aids such as nicotine gum, nicotine patches, electronic cigarette, vapes etc? Prescription, purchased or obtained in other ways? How did these help, or not? Who helped you or hindered you in quitting?
    - Why did this attempt fail, in your view? (*If only one attempt, and it was successful*):
- Successful attempt: Why was this attempt successful, in your view? Anything different in this quit attempt compared with previous ones? (PROBE on sampling characteristic too; e.g., change in employment status). What factor(s) do you think helped you *the most* to quit smoking? What factor(s) made it hardest to quit?

- Looking back on your successful quit attempt, is there anything you would do differently with the benefit of what you know now? Is there anything that would have made it <u>easier</u> for you?

- Do you think you will smoke tobacco again? Why do you say that?

**Never smoked: 15 mins**

These questions are for those people who have never smoked. We are interested in understanding why they did not take up smoking when others around them did/do smoke. Can use the ***Timeline*** *again, particularly* ***Column C*** *(but also* ***Column B****) as individuals may have felt pressured to smoke at different times, or may have been tempted.*

Reasons for not smoking tobacco, the type of questions you can ask here are:

- Has there ever been anytime in your life when you have been tempted to smoke? Probe fully on this. ? Were you tempted to smoke cigarettes at all? When was that and tell me a bit about that situation?
    - Who was there with you, if anyone
    - What were you doing.
    - How old were you
    - Where was it?
      What about smoking anything else? Do you or have you ever tried smoking anything else? Probe fully for details

  Have there been any times you felt pressure to try smoking? Again probe fully for who, what, age, where and why felt this pressure? What did you do instead at that time?

- What, if anything, do you think made you decide *not* to smoke tobacco? What, if anything, put you off smoking? Have these reasons for not smoking changed over time at all? If so, in what way? *Probe on factors such as personal health, cost, dislike of habit (smell etc), illness/death of family member of friend, health promotion, etc*

- Did anyone in your family smoke in the household when you were growing up? Does anyone smoke in your household now?

- Who, if anyone, do you know who smokes around you? Friends and family members? What do you think of this? Have you ever/Do you encourage(d) them to stop? Have any of them managed to quit? If so, what helped them? On the other hand, what has hindered quit attempts? How common is /was smoking in your community ?

- *Repeat questions relating to presence of smoking in their lives from pre -16 up until current age*

- Do you think smoking tobacco is something you will try at some point in the future? Why is that?

ASK ALL: Diary

A TYPICAL DAY (Diary)
I would like you to talk me through a typical day for you nowadays. I would like to understand in detail what you are doing from when you wake up to when you go to bed. So what you do, where you are, who you are with, what you might be feeling at that time.

Smokers- when you smoke (or use e-cigarettes) if at all

Ex-smokers- what you might be feeling at that time, when you used to smoke (or use e-cigarettes) if at all

Never smokers- are with people who are smoking or are exposed to smoking (or using e-cigarettes) if at all.

AN ATYPICAL DAY (Diary)
Now I would like you to talk you through a different type of day. The sort I am thinking of is a weekend day or a day when you have a different pattern/routine. Again, I would like to understand in detail what you did from when you woke up to when you went to bed. So what you did, where you were, who you were with, what you were feeling at that time

Smokers- when you were smoking (or use e-cigarettes) if at all

Ex-smokers- what you might be feeling at that time, when you used to smoke (or use e-cigarettes) if at all

Never smokers- when you were with people who were smoking (or using e-cigarettes) if at all.

We are particularly interested in what you **feel** *about smoking.*
For smokers – *habit, a certain feeling e.g. stress, anxiety, boredom,*
For non-smokers and never smokers *what they do instead of smoking when they have similar feelings to smokers.*

Views of E-cigs/vapes and use – ask all individuals (10-15mins)

*We are also interested in whether you have tried e-cigs/ vaping (***Column D on Timeline***)– (If not already covered) ask questions such as:*

- Are you aware of Ecigs i.e. cigalikes, vapes/vapourisers and box mods? Where do you get your knowledge from in relation to these different devices?
- What do you think of Ecigs, vapes and mods? What are the major pros and cons to them?
- Have you ever tried e-cigs, Vaping, Mods? If tried, do you currently use any of these? Can use **Timeline** to cover use over time
- How often did/do you use them? Do you know others who use them? If so, who? Use **Column E on Typical and Atypical days**
- *If stopped using e-cigs, vaping, mods etc go through process and reasons for stopping*

For everyone: Impact of smoking/not smoking (5 mins)

These questions relate to the perceived impact of smoking, or not smoking, on their lives, to those around them and their health. The sorts of questions you can ask here are:

- What impact, if any, do you think (not) smoking has had on you personally? Any impacts on friends or family members around you? *Probe for perceived negatives and positives on health, finances etc*

- If relevant, imagine if you had not started/started smoking all those years ago, do you think it would have made a difference to your life? If so, in what way?

For everyone: Barriers/Facilitators to smoking 10 mins

This section aims to explore the perceived barriers and facilitators to smoking and differences between age groups – under 16, and 16-24yrs
(if relevant) Questions such as the following are suggested:

More generally, we would like to ask you explore what factors may stop people smoking, either from starting smoking in the first place, or when they are active smokers.

- What do you think the main factors which stop people starting to smoke, or may reduce/stop smokers' tobacco use?
    - Is there any difference in these factors for the following age groups: under 16s, 16-19 years, 20-24 years?

- On the other hand, what do you think the main factors are which make it more likely that people will start to smoke, or may continue/increase smoking?
    - Is there any difference in these factors for the following age groups: under 16s, 16-19 years, 20-24 years?

Any other comments/questions?

- Thank respondents for their time
- Reassure re: confidentiality and ask if there is anything they would not like to be discussed/quoted in the final report
- Ask if it's okay to call back to check some of the details after the interview
- Check if participants have any questions
