# Supplementary material for: “Tell them you smoke, you’ll get more breaks”: a qualitative study of occupational and social contexts of young adult smoking in Scotland
Source: BMJ Open. 2018 Dec 31;8(12):e023951. doi: 10.1136/bmjopen-2018-023951 (PMC6318505; doi:10.1136/bmjopen-2018-023951)
Supplement: Supplementary file 2 [file bmjopen-2018-023951supp002.pdf]

Timeline
Diary: a typical day

**AGE**	**Column A**<br>**School/work/ training/unemp**	**Column B**<br>**Social context/life events**	**Column C**<br>**Smoking history (views and behaviour)**<br>(For non smokers include 2nd hand experience of smoking)	**Column D**<br>**E-Cigs history (views and behaviour)**
Pre 16				
16				
17				
18				
19				
20				
21				
22				
23				
24				

Time	Col A<br>Activity	Col B<br>At<br>Home	Col C<br>Other<br>location	Col D<br>Smoking	Col E<br>E-Cigs	Col F<br>Reasons/influences for<br>smoking/e-cigs (or wanting to)? How feeling?
5.00 am<br>5.30 am						
6.00 am<br>6.30 am						
7.00 am<br>7.30 am						
8.00 am<br>8.30 am						
9.00 am<br>9.30 am						
10.00 am<br>10.30 am						
11.00 am<br>11.30 am						
12 noon<br>12.30 pm						
1.00 pm<br>1.30 pm						
2.00 pm<br>2.30 pm						
3.00 pm<br>3.30 pm						
4.00 pm<br>4.30 pm						
5.00 pm<br>5.30 pm						
6.00 pm<br>6.30 pm						
7.00 pm<br>7.30 pm						
8.00 pm<br>8.30 pm						
9.00 pm<br>9.30 pm						
10.00 pm<br>10.30 pm						
11.00 pm<br>11.30 pm						
12 mid<br>12.30 am						
1.00 am<br>1.30 am						
2.00 am<br>2.30 am						
3.00 am<br>3.30 am						
4.00 am<br>4.30 am						

Diary: Atypical Day

Time	Col A<br>Activity	Col B<br>At<br>Home	Col C<br>Other location	Col D<br>Smoking	Col E<br>E-Cigs	Col F<br>Reasons/influences for<br>smoking/e-cigs (or wanting to)? How feeling?
5.00 am<br>5.30 am						
6.00 am<br>6.30 am						
7.00 am<br>7.30 am						
8.00 am<br>8.30 am						
9.00 am<br>9.30 am						
10.00 am<br>10.30 am						
11.00 am<br>11.30 am						
12 noon<br>12.30 pm						
1.00 pm<br>1.30 pm						
2.00 pm<br>2.30 pm						
3.00 pm<br>3.30 pm						
4.00 pm<br>4.30 pm						
5.00 pm<br>5.30 pm						
6.00 pm<br>6.30 pm						
7.00 pm<br>7.30 pm						
8.00 pm<br>8.30 pm						
9.00 pm<br>9.30 pm						
10.00 pm<br>10.30 pm						
11.00 pm<br>11.30 pm						
12 mid<br>12.30 am						
1.00 am<br>1.30 am						
2.00 am<br>2.30 am						
3.00 am<br>3.30 am						
4.00 am<br>4.30 am						
