# Supplementary material for: “Tell them you smoke, you’ll get more breaks”: a qualitative study of occupational and social contexts of young adult smoking in Scotland
Source: BMJ Open. 2018 Dec 31;8(12):e023951. doi: 10.1136/bmjopen-2018-023951 (PMC6318505; doi:10.1136/bmjopen-2018-023951)
Supplement: Author's manuscript [file bmjopen-2018-023951.draft_revisions.pdf]

BMJ Open is committed to open peer review. As part of this commitment we make the peer review history of every article we publish publicly available.

When an article is published we post the peer reviewers' comments and the authors' responses online. We also post the versions of the paper that were used during peer review. These are the versions that the peer review comments apply to.

The versions of the paper that follow are the versions that were submitted during the peer review process. They are not the versions of record or the final published versions. They should not be cited or distributed as the published version of this manuscript.

BMJ Open is an open access journal and the full, final, typeset and author-corrected version of record of the manuscript is available on our site with no access controls, subscription charges or pay-per-view fees (http://bmjopen.bmj.com).

If you have any questions on BMJ Open's open peer review process please email editorial.bmjopen@bmj.com

BMJ Open

'Tell them you smoke, you'll get more breaks' – a qualitative study of occupational and social contexts of young adult smoking.

Journal:	*BMJ Open*
Manuscript ID	bmjopen-2018-023951
Article Type:	Research
Date Submitted by the Author:	09-May-2018
Complete List of Authors:	Delaney, Hannah; ScotCen Social Research,<br>MacGregor, Andrew; ScotCen Social Research<br>Amos, Amanda; The University of Edinburgh, Centre for Popluation Health Sciences
Keywords:	QUALITATIVE RESEARCH, Young adults, Smoking

SCHOLARONE™
Manuscripts

For peer review only

'*Tell them you smoke, you'll get more breaks*' – a qualitative study of occupational and social contexts of young adult smoking.

Authors:

Hannah Delaney, PhD

ScotCen Social Research

Scotiabank House

6 South Charlotte Street

Edinburgh

EH2 4AW

UK

Andy MacGregor, MSc

ScotCen Social Research

Scotiabank House

6 South Charlotte Street

Edinburgh

EH2 4AW

UK

Amanda Amos, PhD

UKCTAS

Usher Institute of Population Health Sciences and Informatics

University of Edinburgh

For peer review only - http://bmjopen.bmj.com/site/about/guidelines.xhtml

For peer review only

Old Medical School

Teviot Place

Edinburgh

EH8 9AG

UK

Correspondence to:

Hannah Delaney, PhD

ScotCen Social Research

Scotiabank House

6 South Charlotte Street

Edinburgh

EH2 4AW

UK

hannahdelaney_1@hotmail.com

Word Count: 4272

For peer review only

For peer review only - http://bmjopen.bmj.com/site/about/guidelines.xhtml

ABSTRACT

Objective: To explore young adults' perceptions and experiences of smoking and their smoking trajectories in the context of their social and occupational histories and transitions, in a country with advanced tobacco control.

Design: In-depth qualitative interviews using day and life grids to explore participants' smoking behaviour and trajectories in relation to their educational, occupational and social histories and transitions.

Setting: Scotland.

Participants: Fifteen 20-24 year old ever-smokers in 2016-17.

Results: Participants had varied and complex educational/employment histories. Becoming and/or remaining a smoker was often related to social context and educational/occupational transitions. In several contexts smoking, and becoming a smoker, had perceived benefits. These included getting work-breaks and dealing with stress and boredom, which were common in the low paid, unskilled jobs undertaken by participants. In some social contexts smoking was used as a marker of time-out and sociability.

Conclusions: The findings indicate that while increased tobacco control, including smokefree policies, and social disapproval of smoking discourages smoking uptake and increases motivations to quit among young adults, in some social and occupational contexts smoking still has perceived benefits. This helps explain why smoking uptake continues into the mid-20s. It also highlights the importance of policies that reduce the perceived

For peer review only - http://bmjopen.bmj.com/site/about/guidelines.xhtml

desirability of smoking, and that create more positive working environments for young adults which address the types of working hours and conditions that may encourage smoking.

STRENGTHS AND LIMITATIONS OF THIS STUDY

- This is one of the few qualitative studies to explore smoking uptake and trajectories in British young adults.
- We recruited 20-24 year olds with diverse smoking histories, and educational and occupational trajectories.
- The in-depth interviews used both day and life grids to explore current and previous smoking patterns in relation to social, occupational and educational contexts.
- As has been found in previous studies, recruiting a purposive sample in this age group was challenging.

For peer review only

INTRODUCTION

Reducing smoking uptake is a key goal of tobacco control strategies in the UK. Recent governmental action has included: reducing tobacco promotion (banning advertising and point-of-sale displays; standardised packaging), reducing cigarettes' affordability (taxation, banning small packs) and availability (increasing age of sale), increasing awareness of health risks (media campaigns, health warnings), and reducing the social acceptability of smoking (smokefree public places and cars).[1] This has significantly impacted on youth smoking. Smoking prevalence in Scottish 15 year olds halved between 2008 and 2015, from 15% to 7%.[2]

However, smoking uptake in the UK continues into the mid-20s, and the decline in smoking among 16-24 year olds has been less than in younger age groups, from 28% in 2008 to 21% in 2016 in Scotland.[3] This age group is highlighted in the Scottish Tobacco Control Strategy[4] and English Tobacco Plan[5] as being of concern, as over a third of 16-24 year old smokers started smoking when aged 16 or over.[5] Similarly, a US longitudinal study found that 18% of ever-smokers under 30 years started smoking between 18 and 21.[6] Understanding the smoking beliefs, behaviour and social contexts of young adults is vital for developing effective strategies to reduce smoking in this key age group.

Young adulthood can be a time of increased autonomy and freedom to explore different identities and behaviours before more stable roles and responsibilities in later adult life.[7] Young adults often move in and out of smoking[8] and health behaviours can be taken up, consolidated or abandoned.[9,10] This period of fluidity presents an opportunity to prevent never smokers or those who regard themselves as just 'social' smokers, from becoming regular smokers, and to encourage smokers to quit. Qualitative studies have found that identity construction and presentation of self, which Goffman[11] conceptualised as staging a

For peer review only - http://bmjopen.bmj.com/site/about/guidelines.xhtml

performance that is expected in certain situations and that is credited with desired attributes by other actors in that context, are important in understanding young adults' smoking.[12,13,14,15,16] The transitions of young adulthood, which traditionally involve leaving school, leaving the parental home, taking up full-time employment, starting cohabitation and having children,[17] also shape smoking behaviour. Wiltshire et al's[18] study of Scottish 16-19 year olds highlighted the impact of transitions from school to work, further education or unemployment on becoming and staying a smoker. Smoking was perceived by smokers as a lubricant for social relations and a marker of an acceptable identity in certain new occupational or social contexts, which reinforced and increased smoking. Smokers also often associate smoking with drinking, particularly young adults who typically spend more time than older adults socialising with friends in bars, clubs and at parties.[18, 16]

It was expected that the UK's smokefree legislation might particularly impact on this age group through reducing opportunities to smoke in educational, occupational and leisure settings, requiring smokers to go outside to smoke, thereby disrupting the perceived social role and value of smoking in these contexts.[18] This has added importance for smokers who despite often high consumption levels, usually when drinking alcohol, do not regard themselves as 'proper' smokers and risk becoming regular smokers.[19,20] Rooke et al's[21] qualitative study of the impact of the English smokefree legislation, found that it was accepted by young adult smokers as it represented a continuation of smoking denormalisation processes that had characterised their lives. However, smoking remained for them an integral activity of the night-time economy, and a marker of pleasure and sociability. Rooke's study was undertaken 10 years ago following the legislation. It is important to gain a contemporary understanding of how smoking is perceived by young adult smokers. In particular, how the now embedded smoking restrictions and continued denormalisation of smoking are shaping smoking behaviour and trajectories, not least at times of significant life transitions.

For peer review only - http://bmjopen.bmj.com/site/about/guidelines.xhtml

This qualitative study is part of a larger study investigating smoking patterns and trends in 16-24 year olds in Scotland. It aimed to explore, through in-depth interviews, young adults' perceptions and experiences of smoking, and smoking trajectories, in the context of their social and occupational histories/transitions. It also aimed to increase our understanding of why, in a country with the most advanced tobacco control in Europe[22], smoking uptake continues into the mid-twenties.

METHODS

This paper presents the qualitative findings from a mixed methods study involving: (i) secondary analysis of the 2012-15 annual Scottish Health Surveys (SHeS)[3] examining smoking in 16-24 year olds by sociodemographic factors and (ii) qualitative interviews with purposively selected 2014-2015 survey respondents in 2016-17. SHeS is Scotland's most robust national dataset on population health behaviours including smoking.[3]

Purposive sampling of 16-24 year old SHeS respondents was undertaken to recruit participants who had consented to be re-contacted for follow-up research. Preliminary analysis (unpublished) of the 2012-15 survey data showed that smoking prevalence varied by age (16% in 16-19 year olds vs 26% in 20-24 year olds) and economic activity, with 20-24 year olds in full-time education reporting the lowest smoking prevalence (20%), compared with 25% in the employed, and 42% in the NEET (Not in Education, Employment, Training) categories. The purposive sampling aimed to recruit a diverse sample with differing smoking and occupational histories using these criteria: smoking status (ever-smoked), age at qualitative interview (20-24), gender, and economic activity. Invitation letters, information sheets and consent forms were sent to eligible SHeS respondents. Fifteen returned consent forms and were interviewed in December 2016 - April 2017. Participants received a £20 high street voucher.

For peer review only - http://bmjopen.bmj.com/site/about/guidelines.xhtml

Table1. Sample characteristics at the time of the SHeS survey and the qualitative interview

	**SHeS Survey (2014-15)**			**Qualitative Interview (2016-17)**		
	Male	*Female*	*Total*	*Male*	*Female*	*Total*
Current smoker	7	6	13	6	3	9
Ex-regular	1	-	1	2	2	4
Ex-occasional	1	-	1	1	1	2
Education	2	2	4	-	2	2
Employment	6	2	8	9	3	12
NEET	1	2	3	-	1	1
Total	9	6	15	9	6	15

The interviews were conducted face-to-face by two female experienced qualitative researchers in the participants' homes. They were digitally recorded and lasted 1-2 hours. Ethical approval was obtained from NatCen REC. The interviews explored participants' current smoking behaviour and history, occupational and social transitions (e.g. leaving school, un/employment, new social contexts/networks), and the perceived influence of these transitions and contexts on their smoking. The interview used two adapted versions of the 'life grid'. Versions of the life grid have been used in qualitative studies of smoking to collect retrospective smoking histories[23], and current smoking behaviour across the course of a day.[24,25] The first grid, the life grid, recorded structured data on a timeline (before 16 years and each subsequent year) of the participant's work/education, social and smoking history. Key changes and transitions were recorded for each year. The second grid, the day grid, recorded participants' daily routines and smoking behaviour for each hour of a typical and atypical (eg weekend) day. The grids were completed as a joint endeavour between interviewer and participant. They helped build rapport and were often returned to during the interview, allowing interviewer and participant to reflexively refocus and/or elaborate on themes.

For peer review only - http://bmjopen.bmj.com/site/about/guidelines.xhtml

The interviews were transcribed and entered into NVivo 10 to facilitate data management. The transcripts were read in conjunction with the grids to contextualise smoking behaviour and trajectories. Data were analysed thematically, informed by Braun and Clark's[26] phases of thematic analysis. The initial analysis involved familiarisation; transcripts were read and re-read by the co-authors and emergent themes discussed. Codes were systematically compared to identify crosscutting themes and highlight common experiences, as well as differing views. The coding framework was further refined into key themes. Where quotations are used in the Results, participants are identified by a pseudonym, age in years, and smoking status.

Patient involvement: no patients involved in this study.

RESULTS

Participants' employment and smoking status

Participants tended to move from education or unemployment (NEET) into employment between the SHeS survey and the qualitative interview (Table 1). Two participants were now in full-time education, 12 were employed (10 full-time, 2 part-time) and one was still unemployed (NEET).

The interviews revealed varied and complex employment histories, with most of those in employment having had several jobs since leaving school. For instance, since leaving school at 17, Rachel (22, ex-smoker) had had five jobs in three different sectors (hospitality, retail, hairdressing). Participants' current employment sectors included retail, hospitality (e.g. bars, restaurants), skilled manual labour (e.g. painting, roofing) and administration/services (e.g. call centres), with shift work, part-time and zero hour contracts common. Nine participants had attended college or university and two had been apprentices. Only three participants had followed the 'traditional' route of university/college or undertaking an apprenticeship

For peer review only - http://bmjopen.bmj.com/site/about/guidelines.xhtml

followed by full-time employment. Six participants had at some point been unemployed (NEET).

Nine participants were current smokers. Four of the six ex-smokers had quit since the survey (Table 1). Eleven had started smoking before 16, mostly trying their first cigarette between 13 and 15 in social situations. Four participants started smoking later, one at 17 and three at 18. This was typically associated with socialising at parties or clubs when drinking, and starting university/college or a new job. Smoking consumption varied considerably from 3 to 30 cigarettes a day.

While most had smoked for several years, only two described smoking trajectories of increasing consumption since starting to smoke. For other participants, irrespective of the age that they first tried a cigarette, accounts of their smoking histories included at least one attempt to quit (often short-lived), and significant increases and decreases in consumption. Of the six ex-smokers, two were occasional and four were regular smokers before quitting. Some ex-smokers articulated clear reasons for deciding to quit such as significant events (eg a relative's death from a smoking-related illness), while others had more general reasons including no longer finding smoking appealing, wanting to improve their health and finances and their peer group no longer consisting of smokers.

Three themes emerged as important in influencing smoking behaviours and trajectories - occupational role and context, social context, and domestic context.

Occupational smoking

Participants' accounts highlighted that there still appear to be opportunities to smoke, and even to start smoking, at work. As workplaces adhere to smokefree legislation, smokers take breaks outside the office or other enclosed environments. Due to the relatively transient

For peer review only - http://bmjopen.bmj.com/site/about/guidelines.xhtml

nature of occupations in this age group, temporary jobs such as hospitality, retail and shift work were common. For many participants these work contexts were described as encouraging them to move from being non-smokers or social smokers (only borrowing cigarettes from friends), to regular smokers (buying their own cigarettes).

Participants described taking up smoking specifically so that they could take breaks at work. Sarah (22, smoker) who started waitressing at 18 to save money before going travelling, was advised: *'Tell them you smoke. You'll get more breaks'*. She explained how this led to smoking initiation: *'you don't even want a cigarette, but you would just go out and maybe walk around for a bit, and then it got to the point where we would just start having cigarettes'*. Similarly, Jamie (24, ex-smoker) had first tried a cigarette aged 12 but had no interest after that, until he started working at a hotel when 16 and became a regular smoker:

> *'At the hotel you weren't allowed a break unless you were a smoker, so I used to go out with the smokers for like a 5 minute break, multiple times a day, and that's when I'd have one, so that they didn't know that I wasn't a smoker… it's the case where you can't be kind of on a break if you're not having a fag, so, if the manager comes out, you need to be holding one!'*

For participants in more permanent employment, smoking seemed to become rooted in the social aspect of taking work-breaks with colleagues. Daniel (23, smoker) resisted smoking throughout school; however, on starting his first office job at 18 he bought a packet of cigarettes so that he could join his team for a cigarette break. He now smoked 15-20 cigarettes a day:

For peer review only - http://bmjopen.bmj.com/site/about/guidelines.xhtml

'I only had like a couple for the first few weeks…I didn't actually enjoy it when I first started it at all. It was just a case of now and again just going out for the sake of it, or if I'm getting really ticked off at work. But then, after about a few weeks, it was a case of smoking the 10, and then all of a sudden it's the 20'.

Others described their daily smoking pattern at work as being deeply ingrained, often as a stress relief; '*a quick moment of solace to go and bolster yourself*' (Chris, 23, smoker) or as a habit that coincided with breaks. Tom's (20, smoker) smoking consumption was determined by the length of breaks during his call centre shift: *'in the half hour break, you used to have two cigarettes…and then my 15 minute I used to have one'*. Relief from boredom also encouraged smoking: '*It turned into more of a dependency when I was at work and I found myself sort of clock watching, waiting to get to my break' (*Rob, 24, ex-smoker*).* Similarly, Sarah (22, smoker) reported *'smoking more than ever'* due to boredom in her call centre job.

In contrast, some participants had moderated their smoking due to occupational concerns. Stephen (23, smoker) reduced his smoking from over 30 to 10-15 a day while training for a fitness test as part of his Navy application. Sarah (22, smoker) reported never smoking during her student nursing placements, as she did not want to present herself to patients as a smoker:

'I really didn't like smoking on placements: coming back and dealing with patients, just stinking of fags. I just don't think it's that professional'.

Similarly, Tom (20, smoker) explained that as his colleagues did not smoke, he didn't want to '*come across like smelly'* and so masked the smell to present himself in an acceptable manner to them and customers at the cinema where he worked;

For peer review only - http://bmjopen.bmj.com/site/about/guidelines.xhtml

'Like I can smell it off me sometimes, so I have like a can o' deodorant in the locker room. Chewing gum, mints. Something like that basically…coz I'm dealing wi' customers an' that…so it's nice to be…like have fresh breath, when I'm talking to people, come across a certain way... presentable to the public basically'.

For several participants their smoking reduced following changes in social groups, often due to occupational or educational transitions. Jamie's (24, ex-smoker) first quit attempt was during his first six months of university due to his new peers being non-smokers. Similarly, Kate (23, ex-smoker) smoked 20 cigarettes a day but this reduced to 6 when she started a new college course with a new group of non-smoking peers;

'Different course, different people, most of the people on this course don't actually smoke. So it's not very nice just having to go out by myself for a fag. You want to quickly smoke your fag rather than take your time and talk to people so it became about 6 a day on this course '.

Smoking and drinking

All participants reported being exposed to smoking when out socially, particularly outside bars and clubs. Smoking increased when drinking alcohol, with several reporting that currently or previously they only smoked when drinking at parties, bars or clubs.

'I smoke quite a lot if I'm out drinking or whatever, but, apart from that, not really' (Duncan, 24, smoker).

This could create conflicting self-identities between the weekday 'structured/controlled' smoker and the weekend 'uncontrolled' social smoker. For example, on weekdays Stephen

For peer review only - http://bmjopen.bmj.com/site/about/guidelines.xhtml

(23, smoker) reported smoking 10 cigarettes across the day; whereas when drinking at the weekend he went outside to the smoking area every half an hour, each time '*smoking two or three'*.

Several participants, including three of the four who started after the age of 16, traced starting smoking to 'social' smoking while drinking. Sarah (22, smoker) recalled how her regular smoking began at 18 during the summer when she was drinking and socialising a lot. Similarly, Louise (22, smoker) explained how social smoking while drinking developed into regular smoking*:*

> *'It was really just a gradual kind of increase, it wasn't any kind of event that said I'm going to smoke more today. I think going out and going to clubs and having a drink and everything does kind of make you smoke more'.*

Other participants discussed their quit attempts and highlighted drinking and socialising as triggering relapse. Tom (20, smoker) talked about struggling to avoid the '*fall back'* into smoking when drinking. Others discussed similar situations, with some cutting down or stopping smoking during the week but '*weekend wise probably still sticking at the same [smoking levels]'* (Rob, 24, ex-smoker).

Smoking and living circumstances

Participants talked about phases when their smoking increased or decreased due to changed social and living circumstances, reflecting the often transient nature of their lives. Seven currently lived independently in rented accommodation and others had had periods away from home, for example, at university. For most, the initial transition to independent living occurred during their late teens and often coincided with increased socialising and drinking, changes in social circles and more control over their finances; which were associated with

For peer review only - http://bmjopen.bmj.com/site/about/guidelines.xhtml

increased smoking. Rachel (22, ex-smoker) explained that she *'went a bit wild'* when she first left home and her smoking increased (due to her relationship break-up):

> *'I'd just split up with my boyfriend of 3 years…so and because I was out of the house [parent's home]…there was nobody telling me that I couldn't so I just...think I done it [smoked] because I could'.*

Stephen (23, smoker) also explained that when he moved to university:

> *'My parents gave me a budget of £200 a month and I was meant to get a job but, I don't know, I just didn't prioritise things like that. So my £200 budget, I used that to buy cigarettes quite a lot'.*

Several participants had a history of transient living circumstances, including living in temporary locations, with new flatmate/s, or travelling abroad for work or a 'gap' year, which they perceived had affected their smoking. Jamie (24, ex-smoker) had quit smoking for six months but *'things took a turn'* when a flatmate who smoked moved in. He started to go for a cigarette with this flatmate and then '*kinda got the taste back again…and that's when I kinda started buying them again'*. Chris's (23, smoker) smoking increased while working in Germany as tobacco was cheaper there. In contrast, Sarah's (22, smoker) smoking decreased when traveling abroad due to tobacco being harder to access and a changed routine.

Participants who had or were living with partners reported changes in smoking frequency and pattern due to their relationships. Kate (23, ex-smoker) explained that smoking had been both a '*stress release*' from her difficult relationship with her (now) ex-partner, and something that they would do together:

> *'I think because our relationship was quite volatile a lot of the time it was kind of like a stress release, the fags'.*

For peer review only - http://bmjopen.bmj.com/site/about/guidelines.xhtml

Her current partner did not smoke, and consequently she had stopped smoking:

> *'I think it's because like I'm a smoker and my partner is a non-smoker and I was like, you know, it must be so gross…So I was just kind of like...it doesn't seem fair'.*

Similarly, Chris (23, smoker) smoked less around his partner due to health concerns:

> *'He's actually got like not very strong lungs, so I don't tend to smoke around him, and actually… probably having been with him for the last few years maybe my smoking has gone down just because I know that it can affect him more…He'll cough, and it'll make me feel bad, so I won't do it!'*

Participants who had only lived with their parent(s) attributed changes in their smoking status or consumption to factors other than their living circumstances, such as changes in social and employment contexts, as previously described.

DISCUSSION

This paper reports the findings of in-depth interviews with young adults which explored their smoking behaviour and trajectories, in the context of their social and occupational histories since leaving school. It aimed to increase our understanding of why, in a country with strong tobacco control where smoking is increasingly denormalised, smoking uptake continues into the mid-twenties.

The study has shown how young adulthood is a period of considerable flux and transition, where becoming and/or remaining a smoker is often related to social context and the nature of educational and occupational transitions. As was found in research conducted with young adults ten years ago shortly after the smokefree legislation[21], participants accepted that their smoking behaviour was subject to both formal and informal social controls. These included

For peer review only - http://bmjopen.bmj.com/site/about/guidelines.xhtml

legal restrictions on smoking in public places and presenting a socially acceptable image in certain professional and social contexts. Participants talked about, without question, going outside to smoke when at work or pubs and clubs, and not exposing non-smoking partners and friends to their smoking in certain social contexts. The need to appropriately manage their smoker identity in different contexts was most marked in accounts of smoking not being an acceptable part of the performance of a professional self in jobs involving direct contact with the public, such as nursing and the hospitality business. This reflects the increasingly negative social climate around smoking in the UK, with smoking being stigmatised as a marker of low social status[25,27,28] and what Goffman has described as a spoilt, polluted identity.[11] In these contexts young adult smokers' appropriate presentation of self appeared to be paramount. While such social controls and meanings could discourage smoking uptake, reduce consumption and increase motivations to quit, there were other contexts where smoking was perceived more positively.

Despite smoking restrictions in pubs and clubs, smoking in these social contexts was still constructed by many as being sociable and an inherent part of relaxing with friends, drinking alcohol. In these contexts smoking not only increased, which could lead to sustained increased consumption, but could encourage young adults who had not smoked when at school to smoke their first cigarettes. Perhaps, as has been found in other studies, in the belief that 'social smoking' would not lead to regular smoking.[29,30,31] These were also common contexts where quit attempts failed.

Perhaps the most unexpected finding was that young adults taking up jobs with particular employment conditions could lead not only to increased smoking, to deal with stress and boredom, but also to smoking initiation. Several participants described how in certain occupational contexts, notably the hospitality industry and continuous demand jobs such as in call centres, being a smoker carried the significant benefit of being permitted short breaks,

For peer review only - http://bmjopen.bmj.com/site/about/guidelines.xhtml

and in some cases was perceived to be the only way of getting breaks. Such accounts were rare in pre-smokefree studies where taking up smoking on transitioning from school to work was attributed more to 'fitting in' with new colleagues.[18] This finding may partly reflect the more transitional and precarious nature of contemporary young adults' lives, where changes in the employment market during the recession have led to more part-time working and low paid jobs in this age group.[32,33] Also more full-time students in the UK are undertaking part-time or temporary jobs to fund their studies.[34] These tend to be in low paid, low skilled occupations which generally have poorer rights to breaks which can lead to 'role overload' where transitions into adult roles can create stressful demands[10]. Thus while the smokefree legislation and declining smoking prevalence means that young adults may be less exposed to pro-smoking influences at work, in some contexts pressures to smoke or start smoking remain.

Finally, the study reconfirmed the importance of peer influence on smoking trajectories in young adulthood across all social, educational and occupational contexts and transitions.[16,18,21,30] Who participants socialised, worked and/or lived with was perceived as impacting on their smoking status and behaviour. Leaving school for college or employment, and leaving home and gaining more independence goes hand-in-hand with forging new social networks.[18,35] These new peer groups may have different smoking norms which can promote or inhibit smoking.

These qualitative data from a small purposive sample cannot be generalised to the Scottish or UK population. While it was not possible to include all patterns of smoking and occupational trajectories, the participants had diverse smoking histories (e.g. started smoking before/after leaving school, increased and/or decreased their smoking, not/tried to quit), and educational/occupational histories. This was a strength of the study particularly given the

For peer review only - http://bmjopen.bmj.com/site/about/guidelines.xhtml

acknowledged challenges of recruiting young adults to research studies, given their often transient lives and living circumstances. This diversity generated potentially important contemporary insights about how such transitions and contexts can affect smoking. It is possible that other young adults may have different experiences, particularly those from more disadvantaged backgrounds where they may experience fewer transitions and opportunities. There is also a need to explore how young adults who experience similar transitions and influences resist smoking. Further studies should explore these issues with young adults in differing social and economic circumstances.

In conclusion, this study indicates that despite increased tobacco control policies and social disapproval of smoking in the UK, in some social and occupational contexts smoking and having a smoking identity still have perceived positive benefits for young adults. Smoking can be used to deal with occupational pressures through getting 'smoking breaks' and/or perceived relief from stress and boredom which are inherent in many low paid, unskilled jobs undertaken by young adults. In addition, in some social contexts smoking has a positive value and is used as a marker of time-out and sociability. This perhaps helps explain why the decline in smoking in young adults is slower than that in younger adolescents where the more restrictive contexts of school and home environments are barriers to smoking uptake.[18] Our findings show how leaving these environments for more transient, independent lives can facilitate or discourage smoking. They highlight the continuing importance of maintaining smokefree policies to reduce the perceived desirable attributes of smoking. They also have wider implications in relation to the need to create more positive working environments for young adults, which include addressing the culture of smoking breaks and the working hours and conditions that may encourage smoking.

For peer review only - http://bmjopen.bmj.com/site/about/guidelines.xhtml

Acknowledgements

The authors would like to thank all of the participants who gave their time and thoughts to the research, as well as colleagues at ScotCen Social Research; Irene Miller and Susan MacLeod for conducting the interviews, Lesley Birse for research and administrative support, and Stephen Hinchliffe and Laura Brown for data management and analysis.

Contributors

AA and AM conceived the study and designed the methods. AM oversaw participant recruitment. All authors read the transcripts and developed the analysis. HD drafted the manuscript with critical contributions from AA and AM in revised versions.

Competing Interests

The authors have no conflicts of interest to disclose.

Funding

This work was supported by a Cancer Research UK (CRUK) Tobacco Advisory Grant (TAG) award. Grant reference number: A21556.

Data Sharing Statement

No additional data available

For peer review only - http://bmjopen.bmj.com/site/about/guidelines.xhtml

For peer review only

REFERENCES

[1] Royal College of Physicians. *Nicotine without smoke: Tobacco harm reduction*. 2016. https://www.rcplondon.ac.uk/projects/outputs/nicotine-without-smoke-tobacco-harm-reduction-0 last accessed 4 October 2017.

[2] *Scottish Schools Adolescent Lifestyle and Substance Use Survey (SALSUS) 2015*. 2016. http://www.gov.scot/Topics/Research/by-topic/health-community-care/social-research/SALSUS, last accessed: 4 November 2017.

[3] McLean J, Christie S, Gray L. *Scottish Health Survey 2016*. Edinburgh: Scottish Government, 2017. http://www.gov.scot/Publications/2017/10/2970/0 last accessed 4 November 2017.

[4] Scottish Government. *Creating a Tobacco-free Generation: A Tobacco Control Strategy for Scotland*. Edinburgh: Scottish Government, 2013. http://www.gov.scot/resource/0041/00417331.pdf last accessed 4 November 2017.

[5] Department of Health. *Towards a Smokefree Generation: a Tobacco ControlPplan for England.* London: Department of Health, 2017. https://www.gov.uk/government/publications/towards-a-smoke-free-generation-tobacco-control-plan-for-england last accessed 4 November 2017.

[6] Dutra LM, Glantz SA, Lisha NE, Song AV. Beyond experimentation: Five trajectories of cigarette smoking in a longitudinal sample of youth. *PLoS ONE* 2017; 12: e0171808. doi:10.1371/journal.pone.0171808.

[7] Arnett JJ. *Emerging Adulthood: The Winding Road from the Late Teens Through the Twenties*. New York: Oxford University Press, 2014.

[8] Amos A, Hastings G, Angus K, et al. *A Review of Young People and Smoking in England.* Public Health Research Consortium. 2009. http://phrc.lshtm.ac.uk/reports.html last accessed 4 November 2017.

For peer review only - http://bmjopen.bmj.com/site/about/guidelines.xhtml

[9] Sweeting H, Jackson C, Haw S. Changes in the socio-demographic patterning of late adolescent health risk behaviours during the 1990s: analysis of two West of Scotland cohort studies. *BMC Public Health* 2011;11:829. doi: 10.1186/1471-2458-11-829.

[10] Green MJ, Leyland AH, Sweeting H, Benzeval M. Causal effects of transitions to adult roles on early adult smoking and drinking: evidence from three cohorts. *Soc Sci Med* 2017; 187: 193-202. doi: 10.1016/j.socscimed.2017.06.018.

[11] Goffman,E. The Presentation of Self in Everyday Life. Woodstock, NY: Overlook Press 1973.

[12]Fry G, Grogan S, Gough B, Conner M. Smoking in the lived world: how young people make sense of the social role cigarettes play in their lives. *Br J Soc Psy* 2008;47:763–780. doi: 10.1348/014466608X288818.

[13] Haines RJ, Poland BD, Johnson JL. Becoming a ‘real’ smoker: cultural capital in young women’s accounts of smoking and other substance use. *Soc Health Illness* 2009;31:66–80. doi: 10.1111/j.1467-9566.2008.01119.x

[14] Wakefield M, Flay B, Nichter M, Giovino G. Role of the media in influencing trajectories of youth smoking. *Addiction* 2003;98(s1):79–103. doi:10.1046/j.1360-0443.98.s1.6.x

[15] Scheffels J. Stigma, or sort of cool: young adults’ accounts of smoking and identity. *Eur J Cultural Studies* 2009;12:469-486. doi: 10.1177/1367549409342513.

[16] Nichter M, Nichter M, Carkoglu A, Lloyd-Richardson E, TERN. Smoking and drinking among college students: “It’s a package deal”. *Drug Alc Dep* 2010; 106, 16–20. doi: 10.1016/j.drugalcdep.2009.07.025.

For peer review only

For peer review only - http://bmjopen.bmj.com/site/about/guidelines.xhtml

[17] Côté J, Bynner JM. Changes in the transition to adulthood in the UK and Canada: the role of structure and agency in emerging adulthood. *J Youth Studies* 2008; 11: 251-268. doi: 10.1080/13676260801946464.

[18] Wiltshire S, Amos A, Haw S, McNeill A. Image, context and transition: smoking in mid-to-late adolescence. *J Adol* 2005; 28:603–617. doi: 10.1016/j.adolescence.2004.12.005.

[19] Amos A, Wiltshire S, Haw S, McNeill A. Ambivalence and uncertainty: experiences of and attitudes towards addiction and smoking cessation in the mid-to-late teens. *Health Ed Res* 2006; 21, 181-191. doi: 10.1093/her/cyh054.

[20] Guillory J, Lisha N, Lee YO, Ling PM. Phantom smoking among young adult bar patrons. *Tob Control* 2017;26:153-157. doi: 10.1136/tobaccocontrol-2015-052821.

[21] Rooke C, Amos A, Highet G, Hargreaves K. Smoking spaces and practices in pubs, bars and clubs: young adults and the English smokefree legislation. *Health Place* 2013; 19:108–115. doi: 10.1016/j.healthplace.2012.10.009.

[22] Joosens L, Raw M. *The Tobacco Control Scale 2016 in Europe*. Association of European Cancer Societies. 2017. www.tobaccocontrolscale.org/wp-content/uploads/2017/03/TCS-2016-in-Europe-COMPLETE-LoRes.pdf last accessed 4 November 2017.

[23] Parry O, Thomson C, Fowkes FG. Life course data collection: qualitative interviewing using the Life Grid. *Soc Res Online* 1999;4. doi: 10.5153/sro.233.

[24] Bancroft A, Wiltshire S, Parry O, Amos A. 'It's like an addiction first thing … afterwards it's like a habit': daily smoking behaviour among people living in areas of deprivation. *Soc Sci Med* 2003; 56: 1261-1267.

[25] Ritchie D, Amos A, Martin C. Public places after smoke-free - a qualitative exploration of the changes in smoking behaviour. Health Place 2010; 16: 461–469. doi: 10.1016/S0277-9536(02)00124-7.

For peer review only

For peer review only - http://bmjopen.bmj.com/site/about/guidelines.xhtml

[26] Braun V, Clark, V. Using thematic analysis in psychology. *Qualitative Research in Psychology* 2006; 3 (2). Doi: 10.1191/1478088706qp063oa.

[27] Graham H. Smoking, stigma and social class. *J Social Policy* 2012; 41, 83–99.

[28] Farrimond HR, Joffe H. Pollution, peril and poverty: a British study of the stigmatization of smokers. J Comm Applied Soc Psych 2006; 16, 481–491.

[29] Leas EC, Zablocki RW, Edland SD, Al-Delaimy WK. Smokers who report smoking but do not consider themselves smokers: A phenomenon in need of further attention. *Tob Control* 2014: 24, 400-403.

[30] Gray RJ, Hoek J, Edwards R. A qualitative analysis of 'informed choice' among young adult smokers. *Tob Control* 25, 46-51.

[31] Schane RE, Glantz SA, Ling PM. Nondaily and social smoking- an increasingly prevalent pattern. *Arch Intern Med* 2009;169, 1742-1744.

[32] IPPR. *Trends in Part-time and Temporary Work*. 2011. http://www.ippr.org/files/uploadedFiles/pressreleases/Part-time%20and%20temporary%20work%20technical%20briefing.pdf last accessed 5th October 2017.

[33] UK Parliament. *Young People in the Labour Market: Key Issues for the 2015 Parliament.* 2015.https://www.parliament.uk/business/publications/research/key-issues-parliament-2015/work/young-people-in-the-labour-market/ last accessed 5th October 2017

[34] Office for National Statistics. *UK Labour Market: September 2017*. 2017. https://www.ons.gov.uk/employmentandlabourmarket/peopleinwork/employmentandemployeetypes/bulletins/uklabourmarket/september2017#young-people-in-the-labour-market last accessed 5 October 2017.

[35] Bell R, Pavis S, Amos A, Cunningham-Burley S. Continuities and changes: teenage smoking and occupational transition. *J Adol* 1999; 22, 683-694.

For peer review only - http://bmjopen.bmj.com/site/about/guidelines.xhtml

For peer review only

For peer review only - http://bmjopen.bmj.com/site/about/guidelines.xhtml

COREQ (COnsolidated criteria for REporting Qualitative research) Checklist

A checklist of items that should be included in reports of qualitative research. You must report the page number in your manuscript where you consider each of the items listed in this checklist. If you have not included this information, either revise your manuscript accordingly before submitting or note N/A.

Topic	Item No.	Guide Questions/Description	Reported on Page No.
**Domain 1: Research team and reflexivity**			
Personal characteristics			
Interviewer/facilitator	1	Which author/s conducted the interview or focus group?	20
Credentials	2	What were the researcher's credentials? E.g. PhD, MD	1
Occupation	3	What was their occupation at the time of the study?	1
Gender	4	Was the researcher male or female?	8
Experience and training	5	What experience or training did the researcher have?	1
Relationship with participants			
Relationship established	6	Was a relationship established prior to study commencement?	n/a
Participant knowledge of the interviewer	7	What did the participants know about the researcher? e.g. personal goals, reasons for doing the research	7
Interviewer characteristics	8	What characteristics were reported about the inter viewer/facilitator? e.g. Bias, assumptions, reasons and interests in the research topic	n/a
**Domain 2: Study design**			
Theoretical framework			
Methodological orientation and Theory	9	What methodological orientation was stated to underpin the study? e.g. grounded theory, discourse analysis, ethnography, phenomenology, content analysis	9
Participant selection			
Sampling	10	How were participants selected? e.g. purposive, convenience, consecutive, snowball	7
Method of approach	11	How were participants approached? e.g. face-to-face, telephone, mail, email	7
Sample size	12	How many participants were in the study?	8
Non-participation	13	How many people refused to participate or dropped out? Reasons?	n/a
Setting			
Setting of data collection	14	Where was the data collected? e.g. home, clinic, workplace	8
Presence of non-participants	15	Was anyone else present besides the participants and researchers?	n/a
Description of sample	16	What are the important characteristics of the sample? e.g. demographic data, date	7
Data collection			
Interview guide	17	Were questions, prompts, guides provided by the authors? Was it pilot tested?	8
Repeat interviews	18	Were repeat inter views carried out? If yes, how many?	n/a
Audio/visual recording	19	Did the research use audio or visual recording to collect the data?	8
Field notes	20	Were field notes made during and/or after the inter view or focus group?	n/a
Duration	21	What was the duration of the inter views or focus group?	8
Data saturation	22	Was data saturation discussed?	n/a
Transcripts returned	23	Were transcripts returned to participants for comment and/or	n/a

For peer review only

Topic	Item No.	Guide Questions/Description	Reported on Page No.
		correction?	
**Domain 3: analysis and findings**			
Data analysis			
Number of data coders	24	How many data coders coded the data?	9
Description of the coding tree	25	Did authors provide a description of the coding tree?	n/a
Derivation of themes	26	Were themes identified in advance or derived from the data?	9
Software	27	What software, if applicable, was used to manage the data?	9
Participant checking	28	Did participants provide feedback on the findings?	n/a
Reporting			
Quotations presented	29	Were participant quotations presented to illustrate the themes/findings? Was each quotation identified? e.g. participant number	9-16
Data and findings consistent	30	Was there consistency between the data presented and the findings?	9-16
Clarity of major themes	31	Were major themes clearly presented in the findings?	9-16
Clarity of minor themes	32	Is there a description of diverse cases or discussion of minor themes?	n/a

Developed from: Tong A, Sainsbury P, Craig J. Consolidated criteria for reporting qualitative research (COREQ): a 32-item checklist for interviews and focus groups. *International Journal for Quality in Health Care*. 2007. Volume 19, Number 6: pp. 349 – 357

Once you have completed this checklist, please save a copy and upload it as part of your submission. DO NOT include this checklist as part of the main manuscript document. It must be uploaded as a separate file.

For peer review only

BMJ Open

'Tell them you smoke, you'll get more breaks' – a qualitative study of occupational and social contexts of young adult smoking in Scotland.

Journal:	*BMJ Open*
Manuscript ID	bmjopen-2018-023951.R1
Article Type:	Research
Date Submitted by the Author:	24-Sep-2018
Complete List of Authors:	Delaney, Hannah; ScotCen Social Research,<br>MacGregor, Andrew; ScotCen Social Research<br>Amos, Amanda; The University of Edinburgh, Centre for Popluation Health Sciences
**Primary Subject Heading**:	Smoking and tobacco
Secondary Subject Heading:	Qualitative research
Keywords:	QUALITATIVE RESEARCH, Young adults, Smoking

SCHOLARONE™
Manuscripts

'*Tell them you smoke, you'll get more breaks*' – a qualitative study of occupational and social contexts of young adult smoking in Scotland.

Authors:

Hannah Delaney, PhD

ScotCen Social Research

Scotiabank House

6 South Charlotte Street

Edinburgh

EH2 4AW

UK

Andy MacGregor, MSc

ScotCen Social Research

Scotiabank House

6 South Charlotte Street

Edinburgh

EH2 4AW

UK

Amanda Amos, PhD

UKCTAS

Usher Institute of Population Health Sciences and Informatics

University of Edinburgh

For peer review only

For peer review only - http://bmjopen.bmj.com/site/about/guidelines.xhtml

Old Medical School
Teviot Place
Edinburgh
EH8 9AG
UK

Correspondence to:

Hannah Delaney, PhD
ScotCen Social Research
Scotiabank House
6 South Charlotte Street
Edinburgh
EH2 4AW
UK
hannahdelaney_1@hotmail.com

Word Count: 4505

For peer review only

For peer review only - http://bmjopen.bmj.com/site/about/guidelines.xhtml

ABSTRACT

Objective: To explore young adults' perceptions and experiences of smoking and their smoking trajectories in the context of their social and occupational histories and transitions, in a country with advanced tobacco control.

Design: In-depth qualitative interviews using day and life grids to explore participants' smoking behaviour and trajectories in relation to their educational, occupational and social histories and transitions.

Setting: Scotland.

Participants: Fifteen 20-24 year old ever-smokers in 2016-17.

Results: Participants had varied and complex educational/employment histories. Becoming and/or remaining a smoker was often related to social context and educational/occupational transitions. In several contexts smoking, and becoming a smoker, had perceived benefits. These included getting work-breaks and dealing with stress and boredom, which were common in the low paid, unskilled jobs undertaken by participants. In some social contexts smoking was used as a marker of time-out and sociability.

Conclusions: The findings indicate that while increased tobacco control, including smokefree policies, and social disapproval of smoking discourages smoking uptake and increases motivations to quit among young adults, in some social and occupational contexts smoking still has perceived benefits. This finding helps explain why smoking uptake continues into the mid-20s. It also highlights the importance of policies that reduce the

For peer review only - http://bmjopen.bmj.com/site/about/guidelines.xhtml

For peer review only

perceived desirability of smoking, and that create more positive working environments for young adults which address the types of working hours and conditions that may encourage smoking.

STRENGTHS AND LIMITATIONS OF THIS STUDY

- This is one of the few qualitative studies to explore smoking uptake and trajectories in British young adults.
- We recruited 20-24 year olds with diverse smoking histories, and educational and occupational trajectories.
- The in-depth interviews used both day and life grids to explore current and previous smoking patterns in relation to social, occupational and educational contexts.
- As has been found in previous studies, recruiting a purposive sample in this age group was challenging. The time between taking part in the Scottish Health Surveys and the qualitative interviews was 1-3 years, increasing the likelihood of changed addresses and phone numbers.

For peer review only

For peer review only - http://bmjopen.bmj.com/site/about/guidelines.xhtml

INTRODUCTION

Reducing smoking uptake is a key goal of tobacco control strategies. In the UK recent governmental action has included: reducing tobacco promotion (banning point-of-sale displays; standardised packaging), reducing cigarettes' affordability (taxation, banning small packs) and availability (increasing age of sale), increasing awareness of health risks (media campaigns, health warnings), and reducing the social acceptability of smoking (smokefree public places and cars).[1] These measures have significantly impacted on youth smoking. Smoking prevalence in Scottish 15 year olds halved between 2008 and 2015, from 15% to 7%.[2]

However, as in many countries[3], smoking uptake in the UK continues into the mid-20s. Also the decline in smoking among 16-24 year olds has been less than in younger age groups, from 28% in 2008 to 21% in 2016 in Scotland.[4] This age group is highlighted in the Scottish Tobacco Control Strategy[5] and English Tobacco Plan[6] as being of concern, as over a third of 16-24 year old smokers started smoking when aged 16 or over.[6] Similarly, a US longitudinal study found that 18% of ever-smokers under 30 years started smoking between 18 and 21[7] and in the European Union 41% of ever-smokers started regular (weekly) smoking between 18 and 25.[3] Understanding the smoking beliefs, behaviour and social contexts of young adults is vital for developing effective strategies to reduce smoking in this key age group.

Young adulthood can be a time of increased autonomy and freedom to explore different identities and behaviours before more stable roles and responsibilities in later adult life.[8] Young adults often move in and out of smoking[9] and health behaviours can be taken up, consolidated or abandoned.[10,11] This period of fluidity presents an opportunity to prevent never smokers or those who regard themselves as 'social' smokers, from becoming regular smokers, and to encourage smokers to quit.[12,13,14] Qualitative studies have found that identity

For peer review only - http://bmjopen.bmj.com/site/about/guidelines.xhtml

construction and presentation of self, which Goffman[15] conceptualised as staging a performance that is expected in certain situations and that is credited with desired attributes by other actors in that context, are important in understanding young adults' smoking.[16,17,18,19,20,21] The transitions of young adulthood, which traditionally involve leaving school, leaving the parental home, taking up full-time employment, starting cohabitation and having children,[22] also shape smoking behaviour. Wiltshire et al's[23] study of Scottish 16-19 year olds highlighted the impact of transitions from school to work, further education or unemployment on becoming and staying a smoker. Smoking was perceived by smokers as a lubricant for social relations and a marker of an acceptable identity in new occupational or social contexts, which reinforced and increased smoking. Studies in the US, New Zealand and UK have found that smoking and drinking are highly associated, particularly among young adults who typically spend more time than older adults socialising with friends in bars, clubs and at parties.[14,20,21,23,24]

It was expected that the UK's smokefree legislation might particularly impact on this age group through reducing opportunities to smoke in educational, occupational and leisure settings, requiring smokers to go outside to smoke, thereby disrupting the perceived social role and value of smoking in these contexts.[23] This change has added importance for smokers who despite often high consumption levels, usually when drinking alcohol, do not regard themselves as 'proper' smokers and risk becoming regular smokers.[12,13,21,25,26] Rooke et al's[27] qualitative study of the English smokefree legislation, found that it was accepted by young adult smokers as it represented a continuation of smoking denormalisation processes that had characterised their lives. However, smoking remained for them an integral activity of the night-time economy, and a marker of pleasure and sociability. This echoes findings from New Zealand where smoking among young adults has moved to the 'liminal' areas outside bars where smoking is permitted.[21,28] Rooke's study was undertaken 10 years ago following

For peer review only - http://bmjopen.bmj.com/site/about/guidelines.xhtml

the legislation. It is important to gain a contemporary understanding of how smoking is perceived by young adult smokers. In particular, how the now embedded smoking restrictions and continued denormalisation of smoking are shaping smoking behaviour and trajectories, not least at times of significant life transitions.

This qualitative study is part of a larger study investigating smoking patterns and trends in 16-24 year olds in Scotland. It aimed to explore, through in-depth interviews, young adults' perceptions and experiences of smoking, and smoking trajectories, in the context of their social and occupational histories/transitions. It also aimed to increase our understanding of why, in a country with the most advanced tobacco control in Europe[29], smoking uptake continues into the mid-twenties.

METHODS

This paper presents the qualitative findings from a mixed methods study involving: (i) secondary analysis of the 2012-15 annual Scottish Health Surveys (SHeS)[4] examining smoking in 16-24 year olds by sociodemographic factors and (ii) qualitative interviews with purposively selected 2014-2015 survey respondents in 2016-17. SHeS is Scotland's most robust national dataset on smoking.[4]

Purposive sampling of 16-24 year old SHeS respondents was undertaken to recruit participants who had consented to be re-contacted for follow-up research. Preliminary analysis (Scottish Health Survey dataset, 2012-2015) of the 2012-15 survey data showed that smoking prevalence varied by age (16% in 16-19 year olds vs 26% in 20-24 year olds) and economic activity, with 20-24 year olds in full-time education reporting the lowest smoking prevalence (20%), compared with 25% in the employed, and 42% in the NEET (Not in Education, Employment, Training) categories. The sampling aimed to recruit a diverse sample with differing smoking and occupational histories using these criteria: smoking status

For peer review only - http://bmjopen.bmj.com/site/about/guidelines.xhtml

(ever-smoked), age at qualitative interview (20-24), gender, and economic activity. Invitation letters, information sheets and consent forms were sent to 85 eligible SHeS respondents. Fifteen returned consent forms and were interviewed in December 2016 - April 2017. Recruiting young adults into studies is difficult as they frequently change addresses.[14] Additionally, although most SHeS households provided a phone number, this was often not the young person's phone number. The time between taking part in SHeS and qualitative interviews was 1-3 years, increasing the likelihood of changed addresses and phone numbers. Only 13 individuals refused to be interviewed. Nine invitations were returned as 'no longer at this address'. In 48 cases there was no response to invitation letters and up to five calls and texts. Participants received a £20 high street voucher.

Table 1. Sample characteristics at the time of the SHeS survey and the qualitative interview

	**SHeS Survey (2014-15)**			**Qualitative Interview (2016-17)**		
	Male	*Female*	*Total*	*Male*	*Female*	*Total*
Current smoker	7	6	13	6	3	9
Ex-regular	1	-	1	2	2	4
Ex-occasional	1	-	1	1	1	2
Education	2	2	4	-	2	2
Employment	6	2	8	9	3	12
NEET	1	2	3	-	1	1
Total	9	6	15	9	6	15

Table 2. Smoking history of interview respondents

Name*	***Age***	***Age first smoked***	***Smoking history***	***Current smoking status***
Adam	23	14	Quit for 3 months at 19	Current smoker (30/day)
Rachel	22	13	Quit at 16, relapsed at 19, quit at 20	Ex-regular smoker
Tom	20	10	Quit at 14, relapsed at 16, cut down recently	Current smoker (3-5/day)
Sarah	22	18	Quit for 3 months at 19	Current smoker (5-7/day)

For peer review only - http://bmjopen.bmj.com/site/about/guidelines.xhtml

Louise	22	18	Trying to cut down and quit	Current smoker (10 /day)
Daniel	23	17	Quit attempt at 20	Current smoker (15-20/day)
Jamie	24	12	Quit for 6 months at 19, cut down with e-cig at 22	Ex-regular smoker
Duncan	23	12	Quit for 1 week at 18	Current smoker (12/ day)
Peter	24	<10	Quit at 17	Ex-occasional smoker
Kate	23	13	Quit attempt at 20 post-pregnancy	Current smoker (+6/day) and uses e-cigarette
Rob	24	17	Quit for 2 days at 21, cut down/quit at 24	Ex-regular smoker and uses e-cigarette
Chris	23	14	Quit for 6 months at 19	Current smoker (4-5/day)
Stephen	23	15	Cut down recently	Current smoker (10/ day)
Alison	21	18	Very low occasional smoker	Ex-occasional smoker
Helen	23	12/13	Quit for 3 months at 21, cut down recently	Current smoker (8-10/day)

*All names are pseudonyms

The interviews were conducted by two female experienced qualitative researchers in participants' homes. They were digitally recorded and lasted 1-2 hours. Ethical approval was obtained from NatCen REC. Interviews explored participants' current smoking behaviour and history, occupational and social transitions (e.g. leaving school, un/employment, new social contexts/networks), and the perceived influence of these transitions and contexts on their smoking. The interview used two adapted versions of the 'life grid' (supplementary file). Versions of the life grid have been used in qualitative studies of smoking to collect retrospective smoking histories[30], and current smoking behaviour across the course of a day.[31,32] The life grid, recorded structured data on a timeline (before 16 years and each subsequent year) of the participant's work/education, social and smoking history. Key changes and transitions were recorded for each year. The day grid recorded participants' daily routines and smoking behaviour for each hour of a typical and atypical (eg weekend) day. The grids were completed as a joint endeavour between interviewer and participant. They helped build rapport and were often returned to during the interview, allowing interviewer and participant to reflexively refocus and/or elaborate on themes.

For peer review only - http://bmjopen.bmj.com/site/about/guidelines.xhtml

The interviews were transcribed and entered into NVivo 10 to facilitate data management. The transcripts were read in conjunction with the grids to contextualise smoking behaviour and trajectories. Data were analysed thematically, informed by Braun and Clark's[33] phases of thematic analysis. The initial analysis involved familiarisation; transcripts were read and re-read by the co-authors and emergent themes discussed. Codes were systematically compared to identify crosscutting themes and highlight common experiences, as well as differing views. The coding framework was further refined into key themes. Where quotations are used in the Results, participants are identified by a pseudonym, age in years, and smoking status.

Patient and public involvement: no patient and public involvement

RESULTS

Participants' employment and smoking status

Participants tended to move from education or unemployment (NEET) into employment between the SHeS survey and the qualitative interview (Table 1). Two participants were now in full-time education, 12 were employed (10 full-time, 2 part-time) and one was still unemployed.

The interviews revealed varied and complex employment histories, with most of those in employment having had several jobs since leaving school. For instance, since leaving school at 17, Rachel (22, ex-smoker) had had five jobs in three different sectors (hospitality, retail, hairdressing). Participants' current employment sectors included retail, hospitality (e.g. bars, restaurants), skilled manual labour (e.g. painting, roofing) and administration/services (e.g. call centres), with shift work, part-time and zero hour contracts common. Nine participants had attended college or university and two had been apprentices. Only three participants had

For peer review only - http://bmjopen.bmj.com/site/about/guidelines.xhtml

followed the ‘traditional’ route of university/college or apprenticeship followed by full-time employment. Six participants had at some point been unemployed for at least several weeks.

Nine participants were current smokers. Four of the six ex-smokers had quit since the survey (Table 1). Eleven had started smoking before 16, mostly trying their first cigarette between 13 and 15 in social situations (Table 2). Four participants started smoking later, one at 17 and three at 18. This was typically associated with socialising at parties or clubs when drinking, and starting university/college or a new job. Smoking consumption varied considerably from 3 to 30 cigarettes a day.

While most had smoked for several years, only two described trajectories of increasing consumption since starting smoking. For other participants, irrespective of age of first trying a cigarette, accounts included at least one attempt to quit (often short-lived), and significant increases and decreases in consumption. Of the six ex-smokers, two were occasional and four were regular smokers before quitting. Some articulated clear reasons for deciding to quit such as significant events (eg relative’s death from a smoking-related illness), while others had more general reasons including no longer finding smoking appealing, wanting to improve their health and finances, and their peer group no longer comprising smokers.

Three themes emerged as important in influencing smoking behaviours and trajectories - occupational role and context, social context, and domestic context.

Occupational smoking

Participants’ accounts highlighted that there still appear to be opportunities to smoke, and even to start smoking, at work. As workplaces adhere to smokefree legislation, smokers take breaks outside the office or other enclosed environments. Due to the relatively transient nature of occupations in this age group, temporary jobs such as hospitality, retail and shift

For peer review only - http://bmjopen.bmj.com/site/about/guidelines.xhtml

work were common. Many participants described these work contexts as encouraging them to move from being non-smokers or social smokers (only borrowing cigarettes from friends), to regular smokers (buying their own cigarettes).

Participants described the functional and social benefits of taking up smoking at work, on the one hand to get more breaks and relieve boredom and stress, and on the other as a means of socialising with colleagues. For instance, Sarah (22, smoker) started waitressing at 18 to save money before going travelling and was advised: '*Tell them you smoke. You'll get more breaks'*. She explained how this led to smoking initiation: *'you don't even want a cigarette, but you would just go out and maybe walk around for a bit, and then it got to the point where we would just start having cigarettes'*. Similarly, Jamie (24, ex-smoker) had first tried a cigarette aged 12 but had no interest after that, until he started working at a hotel when 16 and became a regular smoker:

> *'At the hotel you weren't allowed a break unless you were a smoker, so I used to go out with the smokers for like a 5 minute break, multiple times a day, and that's when I'd have one, so that they didn't know that I wasn't a smoker… you can't be kind of on a break if you're not having a fag, so, if the manager comes out, you need to be holding one!'*

Others described their daily smoking pattern at work as being deeply ingrained, often as a stress relief; '*a quick moment of solace to go and bolster yourself'* (Chris, 23, smoker) or as a habit that coincided with breaks. Tom's (20, smoker) smoking consumption was determined by the length of breaks during his call centre shift: *'in the half hour break, you used to have two cigarettes…and then my 15 minute I used to have one'*. Relief from boredom also encouraged smoking: '*It turned into more of a dependency when I was at work and I found*

For peer review only - http://bmjopen.bmj.com/site/about/guidelines.xhtml

*myself sort of clock watching, waiting to get to my break' (*Rob, 24, ex-smoker*).* Similarly, Sarah (22, smoker) reported *'smoking more than ever'* due to boredom in her call centre job.

For those in more permanent employment, the perceived benefit of smoking seemed to become rooted in the social aspect of taking work-breaks with colleagues. Daniel (23, smoker) resisted smoking throughout school; however, on starting his first office job at 18 he bought a packet of cigarettes so that he could join his team for a cigarette break. He now smoked 15-20 a day:

> *'I only had like a couple for the first few weeks…I didn't actually enjoy it when I first started it at all. It was just a case of now and again just going out for the sake of it, or if I'm getting really ticked off at work. But then, after about a few weeks, it was a case of smoking the 10, and then all of a sudden it's the 20'.*

In contrast to the perceived benefits of smoking at work, others described how they moderated their smoking due to concerns around professional competency, acceptability and demeanour. Stephen (23, smoker), reduced his smoking from over 30 to 10-15 a day while training for a fitness test as part of his Navy application. Sarah (22, smoker) reported never smoking during her student nursing placements, as she did not want to present herself to patients as a smoker:

> *'I really didn't like smoking on placements: coming back and dealing with patients, just stinking of fags. I just don't think it's that professional'.*

For peer review only - http://bmjopen.bmj.com/site/about/guidelines.xhtml

Tom (20, smoker) explained that as his colleagues did not smoke, he didn't want to '*come across like smelly'* and so masked the smell to present himself in an acceptable manner to them and customers at the cinema where he worked;

> *'Like I can smell it off me sometimes, so I have like a can o' deodorant in the locker room. Chewing gum, mints. Something like that basically…coz I'm dealing wi' customers an' that…so it's nice to be…like have fresh breath, when I'm talking to people, come across a certain way... presentable to the public basically'.*

Similarly, several participants described how the perceived social benefits of smoking at work could diminish following changes in social groups due to occupational or educational transitions, resulting in reduced smoking or quitting. Jamie's (24, ex-smoker) first quit attempt was during his first six months of university due to his new peers being non-smokers. Similarly, Kate (23, ex-smoker) smoked 20 cigarettes a day but this reduced to 6 when she started a college course with a new group of non-smoking peers;

> *'Different course, different people, most of the people on this course don't actually smoke. So it's not very nice just having to go out by myself for a fag. You want to quickly smoke your fag rather than take your time and talk to people so it became about 6 a day on this course'.*

Smoking and drinking

All participants reported being exposed to smoking when out socially, particularly outside bars and clubs. Smoking increased when drinking alcohol, with several reporting that currently or previously they only smoked when drinking at parties, bars or clubs.

For peer review only - http://bmjopen.bmj.com/site/about/guidelines.xhtml

> *'I smoke quite a lot if I'm out drinking or whatever, but, apart from that, not really' (Duncan, 24, smoker).*

Several participants, including three of the four who started after the age of 16, traced starting smoking to 'social' smoking while drinking. Sarah (22, smoker) recalled how her regular smoking began at 18 during the summer when she was drinking and socialising a lot. Similarly, Louise (22, smoker) explained how social smoking while drinking developed into regular smoking:

> *'It was really just a gradual kind of increase, it wasn't any kind of event that said I'm going to smoke more today. I think going out and going to clubs and having a drink and everything does kind of make you smoke more'.*

Other participants discussed their quit attempts and highlighted drinking and socialising as triggering relapse. Tom (20, smoker) talked about struggling to avoid the '*fall back'* into smoking when drinking. Others discussed similar situations, with some cutting down or stopping smoking during the week but '*weekend wise probably still sticking at the same [smoking levels]'* (Rob, 24, ex-smoker).

Smoking and living circumstances

Participants talked about phases when their smoking increased or decreased due to changed social and living circumstances, reflecting the often-transient nature of their lives. Seven currently lived independently in rented accommodation and others had had periods away from home, for example, at university. They discussed changes in their smoking levels due to: moving away from home for the first time and the freedom of having their own space and money, living in other countries where tobacco was more or less accessible and affordable, and the smoking status of who they lived with.

For peer review only - http://bmjopen.bmj.com/site/about/guidelines.xhtml

For most, the transition to independent living occurred during their late teens. This coincided with increased socialising and drinking, changes in social circles and more control over their finances, all of which were associated with increased smoking. Rachel (22, ex-smoker) explained that she *'went a bit wild'* when she first left home and her smoking increased:

> *'I'd just split up with my boyfriend of 3 years…so and because I was out of the house [parent's home]…there was nobody telling me that I couldn't so I just...think I done it [smoked] because I could'.*

Stephen (23, smoker) also explained that when he moved to university:

> *'My parents gave me a budget of £200 a month and I was meant to get a job but, I don't know, I just didn't prioritise things like that. So my £200 budget, I used that to buy cigarettes quite a lot'.*

Several participants had a history of transient living circumstances and perceived that changes in their location had affected their smoking. For instance Chris (23, smoker) reported that his smoking increased when he temporarily re-located to Germany for work, as tobacco was cheaper there. In contrast, Sarah (22, smoker) reported that her smoking decreased when travelling abroad on her 'gap' year due to a changed routine and tobacco being harder to access.

For others, changes in who they lived with affected their smoking. For instance, Jamie (24, ex-smoker) had quit smoking for six months but *'things took a turn'* when a flatmate who smoked moved in. He started to go for a cigarette with this flatmate and then '*kinda got the taste back again…and that's when I kinda started buying them again'*. Participants who had or were living with partners also reported changes in smoking consumption due to their relationships. Kate (23, ex-smoker) explained that smoking had played a big part in her

For peer review only - http://bmjopen.bmj.com/site/about/guidelines.xhtml
For peer review only

relationship with her (now) ex-partner, as it was something that they would do together. Her current partner did not smoke, and consequently she had stopped smoking:

> *'I think it's because like I'm a smoker and my partner is a non-smoker and I was like, you know, it must be so gross…So I was just kind of like...it doesn't seem fair'.*

Similarly, Chris (23, smoker) smoked less around his partner due to health concerns:

> *'He's actually got like not very strong lungs, so I don't tend to smoke around him, and actually… probably having been with him for the last few years maybe my smoking has gone down just because I know that it can affect him more…He'll cough, and it'll make me feel bad, so I won't do it!'*

Participants who had only lived with their parent(s) attributed changes in their smoking status or consumption to factors other than their living circumstances, such as changes in social and employment contexts, as previously described.

DISCUSSION

This paper reports the findings of in-depth interviews with young adults which explored their smoking behaviour and trajectories, in the context of their social and occupational histories since leaving school. It aimed to increase our understanding of why, in a country with strong tobacco control where smoking is increasingly denormalised, smoking uptake continues into the mid-twenties.

The study shows how young adulthood is a period of considerable flux and transition, where becoming and/or remaining a smoker is often related to social context and the nature of educational and occupational transitions. As was found in research conducted with young adults ten years ago shortly after the smokefree legislation[27], participants accepted that their

For peer review only - http://bmjopen.bmj.com/site/about/guidelines.xhtml

smoking behaviour was subject to both formal and informal social controls. These included legal restrictions on smoking in public places and presenting a socially acceptable image in certain professional and social contexts. Participants talked about, without question, going outside to smoke when at work or pubs and clubs, and not exposing non-smoking partners and friends to their smoking in certain social contexts. The need to manage their smoker identity in different contexts was most marked in accounts of smoking not being an acceptable part of the performance of a professional self in jobs involving contact with the public, such as nursing and the hospitality business. This reaction reflects the increasingly negative social climate around smoking in the UK and countries such as New Zealand[22,34] with smoking being stigmatised as a marker of low social status[32,35,36] , what Goffman has described as a spoilt, polluted identity.[15] In these contexts, young adult smokers' appropriate presentation of self appeared to be paramount. While such social controls and meanings could discourage smoking uptake, reduce consumption and increase motivations to quit, there were other contexts where smoking was perceived more positively.

Despite smoking restrictions in pubs and clubs, smoking in these social contexts was construed by many as being sociable and inherent to relaxing with friends, drinking alcohol. This could create conflicting self-identities for participants; between the weekday 'structured/controlled' smoker and the weekend 'uncontrolled' social smoker. As has been found previously, in these contexts smoking not only increased, which could lead to sustained increased consumption, but could encourage young adults who had not smoked when at school to smoke their first cigarettes.[21,24] Perhaps, as has been found in other studies, in the belief that 'social smoking' would not lead to regular smoking or that social smokers could deny their smoker identity .[13,37,38,39,40] These were also common contexts where quit attempts failed.

For peer review only - http://bmjopen.bmj.com/site/about/guidelines.xhtml

Perhaps the most unexpected finding was that young adults taking up jobs with particular employment conditions could lead not only to increased smoking, to deal with stress and boredom, but also to smoking initiation. Several participants described how in certain occupational contexts, notably the hospitality industry and continuous demand jobs in call centres, being a smoker carried the significant benefit of short breaks, and in some cases was the only way of getting breaks. Such accounts were rare in pre-smokefree studies where taking up smoking on transitioning from school to work was attributed more to 'fitting in' with new colleagues.[23] This finding may partly reflect the more transitional and precarious nature of contemporary young adults' lives, where changes in the employment market during the recession have led to more part-time working and low paid jobs in this age group.[41,42] Also more full-time students in the UK are undertaking part-time or temporary jobs to fund their studies.[43] These tend to be in low paid, low skilled occupations which generally have poorer rights which can lead to 'role overload' where transitions into adult roles can create stressful demands[11]. Thus while the smokefree legislation and declining smoking prevalence means that young adults are less exposed to pro-smoking influences at work, in some contexts pressures to smoke or start smoking remain.

Finally, the study reconfirmed the importance of peer influence on smoking trajectories in young adulthood across all social, educational and occupational contexts and transitions.[20,23,27,38] Who participants socialised, worked and/or lived with was perceived as impacting on their smoking status and behaviour. Leaving school for college or employment, and leaving home and gaining more independence goes hand-in-hand with forging new social networks[23,44] , which may have different smoking norms which promote or inhibit smoking.

These qualitative data from a small purposive sample cannot be generalised to the Scottish population. While it was not possible to include all patterns of smoking and occupational

For peer review only - http://bmjopen.bmj.com/site/about/guidelines.xhtml

trajectories, the participants had diverse smoking histories (e.g. started smoking before/after leaving school, increased and/or decreased their smoking, not/tried to quit), and educational/occupational histories. This diversity was a strength of the study particularly given the acknowledged challenges of recruiting young adults to research studies, given their often transient lives and living circumstances.[14] This diversity generated potentially important contemporary insights about how such transitions and contexts can affect smoking. It is possible that other young adults may have different experiences, particularly those from more disadvantaged backgrounds where they may experience fewer transitions and opportunities. There is also a need to explore how young adults experiencing similar transitions and influences resist smoking. Further studies should explore these issues with young adults in differing social and economic circumstances.

In conclusion, this study indicates that despite increased tobacco control policies and social disapproval of smoking, in some social and occupational contexts smoking and having a smoking identity still have perceived positive benefits for young adults. Smoking can be used to deal with occupational pressures through getting 'smoking breaks' and/or perceived relief from stress and boredom which are inherent in many low paid, unskilled jobs undertaken by young adults. In addition, in some social contexts smoking is used as a marker of time-out and sociability. This perhaps helps explain why the decline in young adults' smoking is slower than that in younger adolescents where the more restrictive contexts of school and home environments are barriers to smoking uptake.[23] Our findings show how leaving these environments for more transient, independent lives can facilitate or discourage smoking. They highlight the continuing importance of maintaining smokefree policies to reduce the perceived desirable attributes of smoking. They also have wider implications in relation to the need to create more positive working environments for young adults, which include working with employers, employer organisations and unions to address the culture of smoking breaks

For peer review only - http://bmjopen.bmj.com/site/about/guidelines.xhtml

and the working hours and conditions that encourage smoking. Finally, the strong link between smoking and alcohol highlights the challenge of how to decouple further drinking from smoking, perhaps through requiring completely smokefree areas outside bars, a proposal that has found support among social smokers in New Zealand.[21] However, it also presents the opportunity of targeting socioculturally tailored smoking interventions to young adults in bars and clubs, an approach that has produced promising results in the US.[14,45,46]

Acknowledgements

The authors would like to thank all of the participants who gave their time and thoughts to the research, as well as colleagues at ScotCen Social Research; Irene Miller and Susan MacLeod for conducting the interviews, Lesley Birse for research and administrative support, and Stephen Hinchliffe and Laura Brown for data management and analysis.

Contributors

AA and AM conceived the study and designed the methods. AM oversaw participant recruitment. All authors read the transcripts and developed the analysis. HD drafted the manuscript with critical contributions from AA and AM in revised versions.

Competing Interests

The authors have no conflicts of interest to disclose.

Funding

For peer review only

For peer review only - http://bmjopen.bmj.com/site/about/guidelines.xhtml

This work was supported by a Cancer Research UK (CRUK) Tobacco Advisory Grant (TAG) award. Grant reference number: A21556.

Data Sharing Statement

No additional data available

REFERENCES

[1] Royal College of Physicians. *Nicotine without smoke: Tobacco harm reduction*. 2016. https://www.rcplondon.ac.uk/projects/outputs/nicotine-without-smoke-tobacco-harm-reduction-0 last accessed 4 October 2017.

[2] *Scottish Schools Adolescent Lifestyle and Substance Use Survey (SALSUS) 2015*. 2016. http://www.gov.scot/Topics/Research/by-topic/health-community-care/social-research/SALSUS, last accessed: 4 November 2017.

[3] European Union. *Special Eurobarometer 458- Attitudes of Europeans towards Tobacco and electronic cigarettes.* 2017. Brussels, European Union. http://ec.europa.eu/COMMFrontOffice/publicopinion/index.cfm last accessed 10 September 2018

[4] McLean J, Christie S, Gray L. *Scottish Health Survey 2016*. Edinburgh: Scottish Government, 2017. http://www.gov.scot/Publications/2017/10/2970/0 last accessed 4 November 2017.

[5] Scottish Government. *Creating a Tobacco-free Generation: A Tobacco Control Strategy for Scotland*. Edinburgh: Scottish Government, 2013. http://www.gov.scot/resource/0041/00417331.pdf last accessed 4 November 2017.

For peer review only - http://bmjopen.bmj.com/site/about/guidelines.xhtml

[6] Department of Health. *Towards a Smokefree Generation: a Tobacco ControlPplan for England.* London: Department of Health, 2017. https://www.gov.uk/government/publications/towards-a-smoke-free-generation-tobacco-control-plan-for-england last accessed 4 November 2017.

[7] Dutra LM, Glantz SA, Lisha NE, Song AV. Beyond experimentation: Five trajectories of cigarette smoking in a longitudinal sample of youth. *PLoS ONE* 2017; 12: e0171808. doi:10.1371/journal.pone.0171808.

[8] Arnett JJ. *Emerging Adulthood: The Winding Road from the Late Teens Through the Twenties*. New York: Oxford University Press, 2014.

[9] Amos A, Hastings G, Angus K, et al. *A Review of Young People and Smoking in England.* Public Health Research Consortium. 2009. http://phrc.lshtm.ac.uk/reports.html last accessed 4 November 2017.

[10] Sweeting H, Jackson C, Haw S. Changes in the socio-demographic patterning of late adolescent health risk behaviours during the 1990s: analysis of two West of Scotland cohort studies. *BMC Public Health* 2011;11:829. doi: 10.1186/1471-2458-11-829.

[11] Green MJ, Leyland AH, Sweeting H, Benzeval M. Causal effects of transitions to adult roles on early adult smoking and drinking: evidence from three cohorts. *Soc Sci Med* 2017; 187: 193-202. doi: 10.1016/j.socscimed.2017.06.018.

[12] Song A, Ling P. Social smoking among young adults: investigation of intentions and attempts to quit. *Am J Public Health* 2011;101, 1291 -1296.

[13] Kingsbury JH, Parks MJ, Amato MS, Boyle RG. Deniers and admitters: examining smoker identities in a changing tobacco landscape. *Nicotine Tob Res* 2016;18, 2130-37.

For peer review only - http://bmjopen.bmj.com/site/about/guidelines.xhtml

[14] Ling PM, Holmes LM, Jordan JW, Lisha NE, Bibbins-Domingo K. Bars, nightclubs, and cancer prevention: new approaches to reduce young adult cigarette smoking. *Am J Prev Med* 2017; 53, S78-S85.

[15] Goffman,E. The Presentation of Self in Everyday Life. Woodstock, NY: Overlook Press 1973.

[16]Fry G, Grogan S, Gough B, Conner M. Smoking in the lived world: how young people make sense of the social role cigarettes play in their lives. *Br J Soc Psy* 2008;47:763–780. doi: 10.1348/014466608X288818.

[17] Haines RJ, Poland BD, Johnson JL. Becoming a 'real' smoker: cultural capital in young women's accounts of smoking and other substance use. *Soc Health Illness* 2009;31:66–80. doi: 10.1111/j.1467-9566.2008.01119.x

[18] Wakefield M, Flay B, Nichter M, Giovino G. Role of the media in influencing trajectories of youth smoking. *Addiction* 2003;98(s1):79–103. doi:10.1046/j.1360-0443.98.s1.6.x

[19] Scheffels J. Stigma, or sort of cool: young adults' accounts of smoking and identity. *Eur J Cultural Studies* 2009;12:469-486. doi: 10.1177/1367549409342513.

[20] Nichter M, Nichter M, Carkoglu A, Lloyd-Richardson E, TERN. Smoking and drinking among college students: "It's a package deal". *Drug Alc Dep* 2010; 106, 16–20. doi: 10.1016/j.drugalcdep.2009.07.025.

[21] Hoek J, Maubach N, Stevenson R, Gendall P, Edwards R. Social smokers' management of conflicted identities. *Tob Control* 2013;22, 261-5.

[22] Côté J, Bynner JM. Changes in the transition to adulthood in the UK and Canada: the role of structure and agency in emerging adulthood. *J Youth Studies* 2008; 11: 251-268. doi: 10.1080/13676260801946464.

For peer review only

For peer review only - http://bmjopen.bmj.com/site/about/guidelines.xhtml

[23] Wiltshire S, Amos A, Haw S, McNeill A. Image, context and transition: smoking in mid-to-late adolescence. *J Adol* 2005; 28:603–617. doi: 10.1016/j.adolescence.2004.12.005.

[24] Marsh L, Cousins K, Connor J, Kypri K, Gray A, Hoek J. The association of smoking with drinking pattern may provide opportunities to reduce smoking among students. *Kotuitui: NZ J Soc Sci Online* 2016; 11, 72-81.

[25] Amos A, Wiltshire S, Haw S, McNeill A. Ambivalence and uncertainty: experiences of and attitudes towards addiction and smoking cessation in the mid-to-late teens. *Health Ed Res* 2006; 21, 181-191. doi: 10.1093/her/cyh054.

[26] Guillory J, Lisha N, Lee YO, Ling PM. Phantom smoking among young adult bar patrons. *Tob Control* 2017;26:153-157. doi: 10.1136/tobaccocontrol-2015-052821.

[27] Rooke C, Amos A, Highet G, Hargreaves K. Smoking spaces and practices in pubs, bars and clubs: young adults and the English smokefree legislation. *Health Place* 2013; 19:108–115. doi: 10.1016/j.healthplace.2012.10.009.

[28] Gifford H, Tautolo D, Erick S, Hoek J, Edwards R, Gray R. A qualitative analysis of Māori and Pacific smokers' views on informed choice and smoking. *BMJ Open* 2016: 6, e011415.

[29] Joosens L, Raw M. *The Tobacco Control Scale 2016 in Europe*. Association of European Cancer Societies. 2017. www.tobaccocontrolscale.org/wp-content/uploads/2017/03/TCS-2016-in-Europe-COMPLETE-LoRes.pdf last accessed 4 November 2017.

[30] Parry O, Thomson C, Fowkes FG. Life course data collection: qualitative interviewing using the Life Grid. *Soc Res Online* 1999;4. doi: 10.5153/sro.233.

[31] Bancroft A, Wiltshire S, Parry O, Amos A. 'It's like an addiction first thing … afterwards it's like a habit': daily smoking behaviour among people living in areas of deprivation. *Soc Sci Med* 2003; 56: 1261-1267.

For peer review only - http://bmjopen.bmj.com/site/about/guidelines.xhtml

[32] Ritchie D, Amos A, Martin C. Public places after smoke-free - a qualitative exploration of the changes in smoking behaviour. *Health Place* 2010; 16: 461–469. doi: 10.1016/S0277-9536(02)00124-7.

[33] Braun V, Clark, V. Using thematic analysis in psychology. *Qualitative Research in Psychology* 2006; 3 (2). Doi: 10.1191/1478088706qp063oa.

[34] McCool J, Hoek J, Edwards R, Thomson G, Gifford H. Crossing the smoking divide for young adults: Expressions of stigma and identity among smokers and non-smokers. *Nicotine Tob Res* 2012;15,552-6.

[35] Graham H. Smoking, stigma and social class. *J Social Policy* 2012; 41, 83–99.

[36] Farrimond HR, Joffe H. Pollution, peril and poverty: a British study of the stigmatization of smokers. J Comm Applied Soc Psych 2006; 16, 481–491.

[37] Leas EC, Zablocki RW, Edland SD, Al-Delaimy WK. Smokers who report smoking but do not consider themselves smokers: A phenomenon in need of further attention. *Tob Control* 2014: 24, 400-403.

[38] Gray RJ, Hoek J, Edwards R. A qualitative analysis of 'informed choice' among young adult smokers. *Tob Control* 25, 46-51.

[39] Schane RE, Glantz SA, Ling PM. Nondaily and social smoking- an increasingly prevalent pattern. *Arch Intern Med* 2009;169, 1742-1744.

[40] Jiang N, Lee YO, Ling PM. Young adult social smokers: Their co-use of tobacco and alcohol, tobacco-related attitudes, and quitting efforts. *Prev Med* 2014;69,166-171.

[41] IPPR. *Trends in Part-time and Temporary Work*. 2011. http://www.ippr.org/files/uploadedFiles/pressreleases/Part-time%20and%20temporary%20work%20technical%20briefing.pdf last accessed 5th October 2017.

For peer review only - http://bmjopen.bmj.com/site/about/guidelines.xhtml

[42] UK Parliament. *Young People in the Labour Market: Key Issues for the 2015 Parliament.* 2015.https://www.parliament.uk/business/publications/research/key-issues-parliament-2015/work/young-people-in-the-labour-market/ last accessed 5th October 2017

[43] Office for National Statistics. *UK Labour Market: September 2017*. 2017. https://www.ons.gov.uk/employmentandlabourmarket/peopleinwork/employmentandemployeetypes/bulletins/uklabourmarket/september2017#young-people-in-the-labour-market last accessed 5 October 2017.

[44] Bell R, Pavis S, Amos A, Cunningham-Burley S. Continuities and changes: teenage smoking and occupational transition. *J Adol* 1999; 22, 683-694.

[45] Fallin A, TorstenRN, Neilands B, Jordan JW, Hong JS, Ling PM. Wreaking "Havoc" on smoking: social branding to reach young adult "Partiers" in Oklahoma. *Am J Prev Med* 2015; 48, S78-S85.

[46] Kalkhoran S, Lisha NE, Torsten RN, Neilands B, Jordan JW, Ling PM. Evaluation of bar and nightclub intervention to decrease young adult amoking in New Mexico. *J Adol* 2016; 59, 222-229.

For peer review only

For peer review only - http://bmjopen.bmj.com/site/about/guidelines.xhtml

Timeline

Diary: a typical day

AGE	Column A<br>School/work/<br>training/unemp	Column B<br>Social context/life events	Column C<br>Smoking history (views and behaviour)<br>(For non smokers include 2nd hand experience of smoking)	Column D<br>E-Cigs history (views and behaviour)
Pre 16				
16				
17				
18				
19				
20				
21				
22				
23				
24				

For peer review only

Time	Col A Activity	Col B At Home	Col C Other location	Col D Smoking	Col E E-Cigs	Col F Reasons/influences for smoking/e-cigs (or wanting to)? How feeling?
5.00 am						
5.30 am						
6.00 am						
6.30 am						
7.00 am						
7.30 am						
8.00 am						
8.30 am						
9.00 am						
9.30 am						
10.00 am						
10.30 am						
11.00 am						
11.30 am						
12 noon						
12.30 pm						
1.00 pm						
1.30 pm						
2.00 pm						
2.30 pm						
3.00 pm						
3.30 pm						
4.00 pm						
4.30 pm						
5.00 pm						
5.30 pm						
6.00 pm						
6.30 pm						
7.00 pm						
7.30 pm						
8.00 pm						
8.30 pm						
9.00 pm						
9.30 pm						
10.00 pm						
10.30 pm						
11.00 pm						
11.30 pm						
12 mid						
12.30 am						
1.00 am						
1.30 am						
2.00 am						
2.30 am						
3.00 am						
3.30 am						
4.00 am						
4.30 am						

For peer review only

Diary: Atypical Day

Time	Col A Activity	Col B At Home	Col C Other location	Col D Smoking	Col E E-Cigs	Col F Reasons/influences for smoking/e-cigs (or wanting to)? How feeling?
5.00 am						
5.30 am						
6.00 am						
6.30 am						
7.00 am						
7.30 am						
8.00 am						
8.30 am						
9.00 am						
9.30 am						
10.00 am						
10.30 am						
11.00 am						
11.30 am						
12 noon						
12.30 pm						
1.00 pm						
1.30 pm						
2.00 pm						
2.30 pm						
3.00 pm						
3.30 pm						
4.00 pm						
4.30 pm						
5.00 pm						
5.30 pm						
6.00 pm						
6.30 pm						
7.00 pm						
7.30 pm						
8.00 pm						
8.30 pm						
9.00 pm						
9.30 pm						
10.00 pm						
10.30 pm						
11.00 pm						
11.30 pm						
12 mid						
12.30 am						
1.00 am						
1.30 am						
2.00 am						
2.30 am						
3.00 am						
3.30 am						
4.00 am						
4.30 am						

For peer review only

COREQ (COnsolidated criteria for REporting Qualitative research) Checklist

A checklist of items that should be included in reports of qualitative research. You must report the page number in your manuscript where you consider each of the items listed in this checklist. If you have not included this information, either revise your manuscript accordingly before submitting or note N/A.

Topic	Item No.	Guide Questions/Description	Reported on Page No.
**Domain 1: Research team and reflexivity**			
Personal characteristics			
Interviewer/facilitator	1	Which author/s conducted the interview or focus group?	20
Credentials	2	What were the researcher's credentials? E.g. PhD, MD	1
Occupation	3	What was their occupation at the time of the study?	1
Gender	4	Was the researcher male or female?	8
Experience and training	5	What experience or training did the researcher have?	1
Relationship with participants			
Relationship established	6	Was a relationship established prior to study commencement?	n/a
Participant knowledge of the interviewer	7	What did the participants know about the researcher? e.g. personal goals, reasons for doing the research	7
Interviewer characteristics	8	What characteristics were reported about the inter viewer/facilitator? e.g. Bias, assumptions, reasons and interests in the research topic	n/a
**Domain 2: Study design**			
Theoretical framework			
Methodological orientation and Theory	9	What methodological orientation was stated to underpin the study? e.g. grounded theory, discourse analysis, ethnography, phenomenology, content analysis	9
Participant selection			
Sampling	10	How were participants selected? e.g. purposive, convenience, consecutive, snowball	7
Method of approach	11	How were participants approached? e.g. face-to-face, telephone, mail, email	7
Sample size	12	How many participants were in the study?	8
Non-participation	13	How many people refused to participate or dropped out? Reasons?	n/a
Setting			
Setting of data collection	14	Where was the data collected? e.g. home, clinic, workplace	8
Presence of non-participants	15	Was anyone else present besides the participants and researchers?	n/a
Description of sample	16	What are the important characteristics of the sample? e.g. demographic data, date	7
Data collection			
Interview guide	17	Were questions, prompts, guides provided by the authors? Was it pilot tested?	8
Repeat interviews	18	Were repeat inter views carried out? If yes, how many?	n/a
Audio/visual recording	19	Did the research use audio or visual recording to collect the data?	8
Field notes	20	Were field notes made during and/or after the inter view or focus group?	n/a
Duration	21	What was the duration of the inter views or focus group?	8
Data saturation	22	Was data saturation discussed?	n/a
Transcripts returned	23	Were transcripts returned to participants for comment and/or	n/a

For peer review only

Topic	Item No.	Guide Questions/Description	Reported on Page No.
		correction?	
**Domain 3: analysis and findings**			
Data analysis			
Number of data coders	24	How many data coders coded the data?	9
Description of the coding tree	25	Did authors provide a description of the coding tree?	n/a
Derivation of themes	26	Were themes identified in advance or derived from the data?	9
Software	27	What software, if applicable, was used to manage the data?	9
Participant checking	28	Did participants provide feedback on the findings?	n/a
Reporting			
Quotations presented	29	Were participant quotations presented to illustrate the themes/findings? Was each quotation identified? e.g. participant number	9-16
Data and findings consistent	30	Was there consistency between the data presented and the findings?	9-16
Clarity of major themes	31	Were major themes clearly presented in the findings?	9-16
Clarity of minor themes	32	Is there a description of diverse cases or discussion of minor themes?	n/a

Developed from: Tong A, Sainsbury P, Craig J. Consolidated criteria for reporting qualitative research (COREQ): a 32-item checklist for interviews and focus groups. *International Journal for Quality in Health Care*. 2007. Volume 19, Number 6: pp. 349 – 357

Once you have completed this checklist, please save a copy and upload it as part of your submission. DO NOT include this checklist as part of the main manuscript document. It must be uploaded as a separate file.

For peer review only

BMJ Open

'Tell them you smoke, you'll get more breaks' – a qualitative study of occupational and social contexts of young adult smoking in Scotland.

Journal:	*BMJ Open*
Manuscript ID	bmjopen-2018-023951.R2
Article Type:	Research
Date Submitted by the Author:	17-Oct-2018
Complete List of Authors:	Delaney, Hannah; ScotCen Social Research,<br>MacGregor, Andrew; ScotCen Social Research<br>Amos, Amanda; The University of Edinburgh, Centre for Popluation Health Sciences
**Primary Subject Heading**:	Smoking and tobacco
Secondary Subject Heading:	Qualitative research
Keywords:	QUALITATIVE RESEARCH, Young adults, Smoking

SCHOLARONE™ Manuscripts

'Tell them you smoke, you'll get more breaks' – a qualitative study of occupational and social contexts of young adult smoking in Scotland.

Authors:

Hannah Delaney, PhD

ScotCen Social Research

Scotiabank House

6 South Charlotte Street

Edinburgh

EH2 4AW

UK

Andrew MacGregor, MSc

ScotCen Social Research

Scotiabank House

6 South Charlotte Street

Edinburgh

EH2 4AW

UK

Amanda Amos, PhD

UKCTAS

Usher Institute of Population Health Sciences and Informatics

University of Edinburgh

For peer review only

For peer review only - http://bmjopen.bmj.com/site/about/guidelines.xhtml

Old Medical School
Teviot Place
Edinburgh
EH8 9AG
UK

Correspondence to:

Amanda Amos, PhD

UKCTAS
Usher Institute of Population Health Sciences and Informatics
University of Edinburgh
Old Medical School
Teviot Place
Edinburgh
EH8 9AG
UK
Email: amanda.amos@ed.ac.uk
Phone: +44 (0)1316503236

Word Count: 4558

For peer review only

ABSTRACT

Objective: To explore young adults' perceptions and experiences of smoking and their smoking trajectories in the context of their social and occupational histories and transitions, in a country with advanced tobacco control.

Design: In-depth qualitative interviews using day and life grids to explore participants' smoking behaviour and trajectories in relation to their educational, occupational and social histories and transitions.

Setting: Scotland.

Participants: Fifteen 20-24 year old ever-smokers in 2016-17.

Results: Participants had varied and complex educational/employment histories. Becoming and/or remaining a smoker was often related to social context and educational/occupational transitions. In several contexts smoking, and becoming a smoker, had perceived benefits. These included getting work-breaks and dealing with stress and boredom, which were common in the low paid, unskilled jobs undertaken by participants. In some social contexts smoking was used as a marker of time-out and sociability.

Conclusions: The findings indicate that while increased tobacco control, including smokefree policies, and social disapproval of smoking discourages smoking uptake and increases motivations to quit among young adults, in some social and occupational contexts smoking still has perceived benefits. This finding helps explain why smoking uptake continues into the mid-20s. It also highlights the importance of policies that reduce the

For peer review only - http://bmjopen.bmj.com/site/about/guidelines.xhtml

For peer review only

perceived desirability of smoking, and that create more positive working environments for young adults which address the types of working hours and conditions that may encourage smoking.

STRENGTHS AND LIMITATIONS OF THIS STUDY

- This is one of the few qualitative studies to explore smoking uptake and trajectories in British young adults.
- We recruited 20-24 year olds with diverse smoking histories, and educational and occupational trajectories.
- The in-depth interviews used both day and life grids to explore current and previous smoking patterns in relation to social, occupational and educational contexts.
- As has been found in previous studies, recruiting a purposive sample in this age group was challenging. The time between taking part in the Scottish Health Surveys and the qualitative interviews was 1-3 years, increasing the likelihood of changed addresses and phone numbers.

For peer review only

INTRODUCTION

Reducing smoking uptake is a key goal of tobacco control strategies. In the UK recent governmental action has included: reducing tobacco promotion (banning point-of-sale displays; standardised packaging), reducing cigarettes' affordability (taxation, banning small packs) and availability (increasing age of sale), increasing awareness of health risks (media campaigns, health warnings), and reducing the social acceptability of smoking (smokefree public places and cars).[1] These measures have significantly impacted on youth smoking. Smoking prevalence in Scottish 15 year olds halved between 2008 and 2015, from 15% to 7%.[2]

However, as in many countries[3], smoking uptake in the UK continues into the mid-20s. Also the decline in smoking among 16-24 year olds has been less than in younger age groups, from 28% in 2008 to 21% in 2016 in Scotland.[4] This age group is highlighted in the Scottish Tobacco Control Strategy[5] and English Tobacco Plan[6] as being of concern, as over a third of 16-24 year old smokers started smoking when aged 16 or over.6 Similarly, a US longitudinal study found that 18% of ever-smokers under 30 years started smoking between 18 and 21[7] and in the European Union 41% of ever-smokers started regular (weekly) smoking between 18 and 25.[3] Understanding the smoking beliefs, behaviour and social contexts of young adults is vital for developing effective strategies to reduce smoking in this key age group.

Young adulthood can be a time of increased autonomy and freedom to explore different identities and behaviours before more stable roles and responsibilities in later adult life.[8] Young adults often move in and out of smoking[9] and health behaviours can be taken up, consolidated or abandoned.[10,11] This period of fluidity presents an opportunity to prevent never smokers or those who regard themselves as 'social' smokers, from becoming regular smokers, and to encourage smokers to quit.[12,13,14] Qualitative studies have found that identity

For peer review only - http://bmjopen.bmj.com/site/about/guidelines.xhtml

construction and presentation of self, which Goffman[15] conceptualised as staging a performance that is expected in certain situations and that is credited with desired attributes by other actors in that context, are important in understanding young adults' smoking.[16,17,18,19,20,21] The transitions of young adulthood, which traditionally involve leaving school, leaving the parental home, taking up full-time employment, starting cohabitation and having children,[22] also shape smoking behaviour. Wiltshire et al's[23] study of Scottish 16-19 year olds highlighted the impact of transitions from school to work, further education or unemployment on becoming and staying a smoker. Smoking was perceived by smokers as a lubricant for social relations and a marker of an acceptable identity in new occupational or social contexts, which reinforced and increased smoking. Studies in the US, New Zealand and UK have found that smoking and drinking are highly associated, particularly among young adults who typically spend more time than older adults socialising with friends in bars, clubs and at parties.[14,20,21,23,24]

It was expected that the UK's smokefree legislation might particularly impact on this age group through reducing opportunities to smoke in educational, occupational and leisure settings, requiring smokers to go outside to smoke, thereby disrupting the perceived social role and value of smoking in these contexts.[23] This change has added importance for smokers who despite often high consumption levels, usually when drinking alcohol, do not regard themselves as 'proper' smokers and risk becoming regular smokers.[12,13,21,25,26] Rooke et al's[27] qualitative study of the English smokefree legislation, found that it was accepted by young adult smokers as it represented a continuation of smoking denormalisation processes that had characterised their lives. However, smoking remained for them an integral activity of the night-time economy, and a marker of pleasure and sociability. This echoes findings from New Zealand where smoking among young adults has moved to the 'liminal' areas outside bars where smoking is permitted.[21,28] Rooke's study was undertaken 10 years ago following

For peer review only - http://bmjopen.bmj.com/site/about/guidelines.xhtml

the legislation. It is important to gain a contemporary understanding of how smoking is perceived by young adult smokers. In particular, how the now embedded smoking restrictions and continued denormalisation of smoking are shaping smoking behaviour and trajectories, not least at times of significant life transitions.

This qualitative study is part of a larger study investigating smoking patterns and trends in 16-24 year olds in Scotland. It aimed to explore, through in-depth interviews, young adults' perceptions and experiences of smoking, and smoking trajectories, in the context of their social and occupational histories/transitions. It also aimed to increase our understanding of why, in a country with the most advanced tobacco control in Europe[29], smoking uptake continues into the mid-twenties.

METHODS

This paper presents the qualitative findings from a mixed methods study involving: (i) secondary analysis of the 2012-15 annual Scottish Health Surveys (SHeS)[4] examining smoking in 16-24 year olds by sociodemographic factors and (ii) qualitative interviews with purposively selected 2014-2015 survey respondents in 2016-17. SHeS is Scotland's most robust national dataset on smoking.[4]

Purposive sampling of 16-24 year old SHeS respondents was undertaken to recruit participants who had consented to be re-contacted for follow-up research. Preliminary analysis (Scottish Health Survey dataset, 2012-2015) of the 2012-15 survey data showed that smoking prevalence varied by age (16% in 16-19 year olds vs 26% in 20-24 year olds) and economic activity, with 20-24 year olds in full-time education reporting the lowest smoking prevalence (20%), compared with 25% in the employed, and 42% in the NEET (Not in Education, Employment, Training) categories. The sampling aimed to recruit a diverse sample with differing smoking and occupational histories using these criteria: smoking status

For peer review only - http://bmjopen.bmj.com/site/about/guidelines.xhtml

(ever-smoked), age at qualitative interview (20-24), gender, and economic activity. Invitation letters, information sheets and consent forms were sent to 85 eligible SHeS respondents. Fifteen returned consent forms and were interviewed in December 2016 - April 2017. Only 13 individuals refused to be interviewed. Nine invitations were returned as 'no longer at this address'. In 48 cases there was no response to invitation letters and up to five calls and texts. Participants received a £20 high street voucher.

Table 1. Sample characteristics at the time of the SHeS survey and the qualitative interview

	**SHeS Survey (2014-15)**			**Qualitative Interview (2016-17)**		
	Male	*Female*	*Total*	*Male*	*Female*	*Total*
Current smoker	7	6	13	6	3	9
Ex-regular	1	-	1	2	2	4
Ex-occasional	1	-	1	1	1	2
Education	2	2	4	-	2	2
Employment	6	2	8	9	3	12
NEET	1	2	3	-	1	1
Total	9	6	15	9	6	15

Table 2. Smoking history of interview respondents

*Name**	*Age*	*Age first smoked*	*Smoking history*	*Current smoking status*
Adam	23	14	Quit for 3 months at 19	Current smoker (30/day)
Rachel	22	13	Quit at 16, relapsed at 19, quit at 20	Ex-regular smoker
Tom	20	10	Quit at 14, relapsed at 16, cut down recently	Current smoker (3-5/day)
Sarah	22	18	Quit for 3 months at 19	Current smoker (5-7/day)
Louise	22	18	Trying to cut down and quit	Current smoker (10 /day)
Daniel	23	17	Quit attempt at 20	Current smoker (15-20/day)
Jamie	24	12	Quit for 6 months at 19, cut down with e-cig at 22	Ex-regular smoker
Duncan	23	12	Quit for 1 week at 18	Current smoker (12/ day)
Peter	24	<10	Quit at 17	Ex-occasional smoker
Kate	23	13	Quit attempt at 20 post-pregnancy	Current smoker (+6/day) and uses e-cigarette

For peer review only - http://bmjopen.bmj.com/site/about/guidelines.xhtml

Rob	24	17	Quit for 2 days at 21, cut down/quit at 24	Ex-regular smoker and uses e-cigarette
Chris	23	14	Quit for 6 months at 19	Current smoker (4-5/day)
Stephen	23	15	Cut down recently	Current smoker (10/ day)
Alison	21	18	Very low occasional smoker	Ex-occasional smoker
Helen	23	12/13	Quit for 3 months at 21, cut down recently	Current smoker (8-10/day)

*All names are pseudonyms

The interviews were conducted by two female experienced qualitative researchers in participants' homes (Interview Guide, supplementary file). They were digitally recorded and lasted 1-2 hours. Ethical approval was obtained from NatCen REC. Interviews explored participants' current smoking behaviour and history, occupational and social transitions (e.g. leaving school, un/employment, new social contexts/networks), and the perceived influence of these transitions and contexts on their smoking. The interview used two adapted versions of the 'life grid' (supplementary file). Versions of the life grid have been used in qualitative studies of smoking to collect retrospective smoking histories[30], and current smoking behaviour across the course of a day.[31,32] The life grid, recorded structured data on a timeline (before 16 years and each subsequent year) of the participant's work/education, social and smoking history. Key changes and transitions were recorded for each year. The day grid recorded participants' daily routines and smoking behaviour for each hour of a typical and atypical (eg weekend) day. The grids were completed as a joint endeavour between interviewer and participant. They helped build rapport and were often returned to during the interview, allowing interviewer and participant to reflexively refocus and/or elaborate on themes.

The interviews were transcribed and entered into NVivo 10 to facilitate data management. The transcripts were read in conjunction with the grids to contextualise smoking behaviour and trajectories. Data were analysed thematically, informed by Braun and Clark's[33] phases of thematic analysis. The initial analysis involved familiarisation; transcripts were read and re-

For peer review only - http://bmjopen.bmj.com/site/about/guidelines.xhtml

read by the co-authors and emergent themes discussed. Codes were systematically compared to identify crosscutting themes and highlight common experiences, as well as differing views. The coding framework was further refined into key themes. While we cannot say that data saturation was reached, given the diverse sample, the participants did discuss similar experiences and their accounts included several overlapping themes. Where quotations are used in the Results, participants are identified by a pseudonym, age in years, and smoking status.

Patient and public involvement: no patient and public involvement

RESULTS

Participants' employment and smoking status

Participants tended to move from education or unemployment (NEET) into employment between the SHeS survey and the qualitative interview (Table 1). Two participants were now in full-time education, 12 were employed (10 full-time, 2 part-time) and one was still unemployed.

The interviews revealed varied and complex employment histories, with most of those in employment having had several jobs since leaving school. For instance, since leaving school at 17, Rachel (22, ex-smoker) had had five jobs in three different sectors (hospitality, retail, hairdressing). Participants' current employment sectors included retail, hospitality (e.g. bars, restaurants), skilled manual labour (e.g. painting, roofing) and administration/services (e.g. call centres), with shift work, part-time and zero hour contracts common. Nine participants had attended college or university and two had been apprentices. Only three participants had followed the 'traditional' route of university/college or apprenticeship followed by full-time employment. Six participants had at some point been unemployed for at least several weeks.

For peer review only - http://bmjopen.bmj.com/site/about/guidelines.xhtml

Nine participants were current smokers. Four of the six ex-smokers had quit since the survey (Table 1). Eleven had started smoking before 16, mostly trying their first cigarette between 13 and 15 in social situations (Table 2). Four participants started smoking later, one at 17 and three at 18. This was typically associated with socialising at parties or clubs when drinking, and starting university/college or a new job. Smoking consumption varied considerably from 3 to 30 cigarettes a day.

While most had smoked for several years, only two described trajectories of increasing consumption since starting smoking. For other participants, irrespective of age of first trying a cigarette, accounts included at least one attempt to quit (often short-lived), and significant increases and decreases in consumption. Of the six ex-smokers, two were occasional and four were regular smokers before quitting. Some articulated clear reasons for deciding to quit such as significant events (eg relative's death from a smoking-related illness), while others had more general reasons including no longer finding smoking appealing, wanting to improve their health and finances, and their peer group no longer comprising smokers.

Three themes emerged as important in influencing smoking behaviours and trajectories - occupational role and context, social context, and domestic context.

Occupational smoking

Participants' accounts highlighted that there still appear to be opportunities to smoke, and even to start smoking, at work. As workplaces adhere to smokefree legislation, smokers take breaks outside the office or other enclosed environments. Due to the relatively transient nature of occupations in this age group, temporary jobs such as hospitality, retail and shift work were common. Many participants described these work contexts as encouraging them to move from being non-smokers or social smokers (only borrowing cigarettes from friends), to regular smokers (buying their own cigarettes).

For peer review only - http://bmjopen.bmj.com/site/about/guidelines.xhtml

Participants described the functional and social benefits of taking up smoking at work, on the one hand to get more breaks and relieve boredom and stress, and on the other as a means of socialising with colleagues. For instance, Sarah (22, smoker) started waitressing at 18 to save money before going travelling and was advised: '*Tell them you smoke. You'll get more breaks'*. She explained how this led to smoking initiation: *'you don't even want a cigarette, but you would just go out and maybe walk around for a bit, and then it got to the point where we would just start having cigarettes'*. Similarly, Jamie (24, ex-smoker) had first tried a cigarette aged 12 but had no interest after that, until he started working at a hotel when 16 and became a regular smoker:

> *'At the hotel you weren't allowed a break unless you were a smoker, so I used to go out with the smokers for like a 5 minute break, multiple times a day, and that's when I'd have one, so that they didn't know that I wasn't a smoker… you can't be kind of on a break if you're not having a fag, so, if the manager comes out, you need to be holding one!'*

Others described their daily smoking pattern at work as being deeply ingrained, often as a stress relief; '*a quick moment of solace to go and bolster yourself'* (Chris, 23, smoker) or as a habit that coincided with breaks. Tom's (20, smoker) smoking consumption was determined by the length of breaks during his call centre shift: *'in the half hour break, you used to have two cigarettes…and then my 15 minute I used to have one'*. Relief from boredom also encouraged smoking: '*It turned into more of a dependency when I was at work and I found myself sort of clock watching, waiting to get to my break' (*Rob, 24, ex-smoker*)*. Similarly, Sarah (22, smoker) reported *'smoking more than ever'* due to boredom in her call centre job.

For peer review only - http://bmjopen.bmj.com/site/about/guidelines.xhtml

For those in more permanent employment, the perceived benefit of smoking seemed to become rooted in the social aspect of taking work-breaks with colleagues. Daniel (23, smoker) resisted smoking throughout school; however, on starting his first office job at 18 he bought a packet of cigarettes so that he could join his team for a cigarette break. He now smoked 15-20 a day:

> *'I only had like a couple for the first few weeks…I didn't actually enjoy it when I first started it at all. It was just a case of now and again just going out for the sake of it, or if I'm getting really ticked off at work. But then, after about a few weeks, it was a case of smoking the 10, and then all of a sudden it's the 20'.*

In contrast to the perceived benefits of smoking at work, others described how they moderated their smoking due to concerns around professional competency, acceptability and demeanour. Stephen (23, smoker), reduced his smoking from over 30 to 10-15 a day while training for a fitness test as part of his Navy application. Sarah (22, smoker) reported never smoking during her student nursing placements, as she did not want to present herself to patients as a smoker:

> *'I really didn't like smoking on placements: coming back and dealing with patients, just stinking of fags. I just don't think it's that professional'.*

Tom (20, smoker) explained that as his colleagues did not smoke, he didn't want to '*come across like smelly'* and so masked the smell to present himself in an acceptable manner to them and customers at the cinema where he worked;

For peer review only - http://bmjopen.bmj.com/site/about/guidelines.xhtml

'Like I can smell it off me sometimes, so I have like a can o' deodorant in the locker room. Chewing gum, mints. Something like that basically…coz I'm dealing wi' customers an' that…so it's nice to be…like have fresh breath, when I'm talking to people, come across a certain way... presentable to the public basically'.

Similarly, several participants described how the perceived social benefits of smoking at work could diminish following changes in social groups due to occupational or educational transitions, resulting in reduced smoking or quitting. Jamie's (24, ex-smoker) first quit attempt was during his first six months of university due to his new peers being non-smokers. Similarly, Kate (23, ex-smoker) smoked 20 cigarettes a day but this reduced to 6 when she started a college course with a new group of non-smoking peers;

'Different course, different people, most of the people on this course don't actually smoke. So it's not very nice just having to go out by myself for a fag. You want to quickly smoke your fag rather than take your time and talk to people so it became about 6 a day on this course'.

Smoking and drinking

All participants reported being exposed to smoking when out socially, particularly outside bars and clubs. Smoking increased when drinking alcohol, with several reporting that currently or previously they only smoked when drinking at parties, bars or clubs.

'I smoke quite a lot if I'm out drinking or whatever, but, apart from that, not really' (Duncan, 24, smoker).

Several participants, including three of the four who started after the age of 16, traced starting smoking to 'social' smoking while drinking. Sarah (22, smoker) recalled how her regular

For peer review only - http://bmjopen.bmj.com/site/about/guidelines.xhtml

smoking began at 18 during the summer when she was drinking and socialising a lot. Similarly, Louise (22, smoker) explained how social smoking while drinking developed into regular smoking:

> *'It was really just a gradual kind of increase, it wasn't any kind of event that said I'm going to smoke more today. I think going out and going to clubs and having a drink and everything does kind of make you smoke more'.*

Other participants discussed their quit attempts and highlighted drinking and socialising as triggering relapse. Tom (20, smoker) talked about struggling to avoid the '*fall back'* into smoking when drinking. Others discussed similar situations, with some cutting down or stopping smoking during the week but '*weekend wise probably still sticking at the same [smoking levels]'* (Rob, 24, ex-smoker).

Smoking and living circumstances

Participants talked about phases when their smoking increased or decreased due to changed social and living circumstances, reflecting the often-transient nature of their lives. Seven currently lived independently in rented accommodation and others had had periods away from home, for example, at university. They discussed changes in their smoking levels due to: moving away from home for the first time and the freedom of having their own space and money, living in other countries where tobacco was more or less accessible and affordable, and the smoking status of who they lived with.

For most, the transition to independent living occurred during their late teens. This coincided with increased socialising and drinking, changes in social circles and more control over their finances, all of which were associated with increased smoking. Rachel (22, ex-smoker) explained that she *'went a bit wild'* when she first left home and her smoking increased:

For peer review only - http://bmjopen.bmj.com/site/about/guidelines.xhtml

'I'd just split up with my boyfriend of 3 years…so and because I was out of the house [parent's home]…there was nobody telling me that I couldn't so I just...think I done it [smoked] because I could'.

Stephen (23, smoker) also explained that when he moved to university:

'My parents gave me a budget of £200 a month and I was meant to get a job but, I don't know, I just didn't prioritise things like that. So my £200 budget, I used that to buy cigarettes quite a lot'.

For peer review only

Several participants had a history of transient living circumstances and perceived that changes in their location had affected their smoking. For instance Chris (23, smoker) reported that his smoking increased when he temporarily re-located to Germany for work, as tobacco was cheaper there. In contrast, Sarah (22, smoker) reported that her smoking decreased when travelling abroad on her 'gap' year due to a changed routine and tobacco being harder to access.

For others, changes in who they lived with affected their smoking. For instance, Jamie (24, ex-smoker) had quit smoking for six months but *'things took a turn'* when a flatmate who smoked moved in. He started to go for a cigarette with this flatmate and then '*kinda got the taste back again…and that's when I kinda started buying them again*'. Participants who had or were living with partners also reported changes in smoking consumption due to their relationships. Kate (23, ex-smoker) explained that smoking had played a big part in her relationship with her (now) ex-partner, as it was something that they would do together. Her current partner did not smoke, and consequently she had stopped smoking:

'I think it's because like I'm a smoker and my partner is a non-smoker and I was like, you know, it must be so gross…So I was just kind of like...it doesn't seem fair'.

For peer review only - http://bmjopen.bmj.com/site/about/guidelines.xhtml

Similarly, Chris (23, smoker) smoked less around his partner due to health concerns:

> *'He's actually got like not very strong lungs, so I don't tend to smoke around him, and actually… probably having been with him for the last few years maybe my smoking has gone down just because I know that it can affect him more…He'll cough, and it'll make me feel bad, so I won't do it!'*

Participants who had only lived with their parent(s) attributed changes in their smoking status or consumption to factors other than their living circumstances, such as changes in social and employment contexts, as previously described.

DISCUSSION

This paper reports the findings of in-depth interviews with young adults which explored their smoking behaviour and trajectories, in the context of their social and occupational histories since leaving school. It aimed to increase our understanding of why, in a country with strong tobacco control where smoking is increasingly denormalised, smoking uptake continues into the mid-twenties.

The study shows how young adulthood is a period of considerable flux and transition, where becoming and/or remaining a smoker is often related to social context and the nature of educational and occupational transitions. As was found in research conducted with young adults ten years ago shortly after the smokefree legislation[27], participants accepted that their smoking behaviour was subject to both formal and informal social controls. These included legal restrictions on smoking in public places and presenting a socially acceptable image in certain professional and social contexts. Participants talked about, without question, going outside to smoke when at work or pubs and clubs, and not exposing non-smoking partners and friends to their smoking in certain social contexts. The need to manage their smoker

For peer review only - http://bmjopen.bmj.com/site/about/guidelines.xhtml

identity in different contexts was most marked in accounts of smoking not being an acceptable part of the performance of a professional self in jobs involving contact with the public, such as nursing and the hospitality business. This reaction reflects the increasingly negative social climate around smoking in the UK and countries such as New Zealand[22,34] with smoking being stigmatised as a marker of low social status[32,35,36] , what Goffman has described as a spoilt, polluted identity.[15] In these contexts, young adult smokers' appropriate presentation of self appeared to be paramount. While such social controls and meanings could discourage smoking uptake, reduce consumption and increase motivations to quit, there were other contexts where smoking was perceived more positively.

Despite smoking restrictions in pubs and clubs, smoking in these social contexts was construed by many as being sociable and inherent to relaxing with friends, drinking alcohol. This could create conflicting self-identities for participants; between the weekday 'structured/controlled' smoker and the weekend 'uncontrolled' social smoker. As has been found previously, in these contexts smoking not only increased, which could lead to sustained increased consumption, but could encourage young adults who had not smoked when at school to smoke their first cigarettes.[21,24] Perhaps, as has been found in other studies, in the belief that 'social smoking' would not lead to regular smoking or that social smokers could deny their smoker identity .[13,37,38,39,40] These were also common contexts where quit attempts failed.

Perhaps the most unexpected finding was that young adults taking up jobs with particular employment conditions could lead not only to increased smoking, to deal with stress and boredom, but also to smoking initiation. Several participants described how in certain occupational contexts, notably the hospitality industry and continuous demand jobs in call centres, being a smoker carried the significant benefit of short breaks, and in some cases was the only way of getting breaks. Such accounts were rare in pre-smokefree studies where

For peer review only - http://bmjopen.bmj.com/site/about/guidelines.xhtml

taking up smoking on transitioning from school to work was attributed more to 'fitting in' with new colleagues.[23] This finding may partly reflect the more transitional and precarious nature of contemporary young adults' lives, where changes in the employment market during the recession have led to more part-time working and low paid jobs in this age group.[41,42] Also more full-time students in the UK are undertaking part-time or temporary jobs to fund their studies.[43] These tend to be in low paid, low skilled occupations which generally have poorer rights which can lead to 'role overload' where transitions into adult roles can create stressful demands[11]. Thus while the smokefree legislation and declining smoking prevalence means that young adults are less exposed to pro-smoking influences at work, in some contexts pressures to smoke or start smoking remain.

Finally, the study reconfirmed the importance of peer influence on smoking trajectories in young adulthood across all social, educational and occupational contexts and transitions.[20,23,27,38] Who participants socialised, worked and/or lived with was perceived as impacting on their smoking status and behaviour. Leaving school for college or employment, and leaving home and gaining more independence goes hand-in-hand with forging new social networks[23,44] , which may have different smoking norms which promote or inhibit smoking.

The main limitation for this study was recruitment, as has been found in previous studies, recruiting young adults into research is difficult as they frequently change addresses given their often transient lives and living circumstances.[14] Additionally, although most SHeS households provided a phone number, this was often not the young person's phone number; the time between taking part in SHeS and the qualitative interviews was 1-3 years, increasing the likelihood of changed addresses and phone numbers. These qualitative data from a small purposive sample cannot be generalised to the Scottish population.

For peer review only - http://bmjopen.bmj.com/site/about/guidelines.xhtml

While it was not possible to include all patterns of smoking and occupational trajectories, the participants had diverse smoking histories (e.g. started smoking before/after leaving school, increased and/or decreased their smoking, not/tried to quit), and educational/occupational histories. This diversity was a strength of the study particularly given the above acknowledged challenges of recruiting young adults to research studies. This diversity generated potentially important contemporary insights about how such transitions and contexts can affect smoking. It is possible that other young adults may have different experiences, particularly those from more disadvantaged backgrounds where they may experience fewer transitions and opportunities. There is also a need to explore how young adults experiencing similar transitions and influences resist smoking. Further studies should explore these issues with young adults in differing social and economic circumstances.

In conclusion, this study indicates that despite increased tobacco control policies and social disapproval of smoking, in some social and occupational contexts smoking and having a smoking identity still have perceived positive benefits for young adults. Smoking can be used to deal with occupational pressures through getting ‘smoking breaks’ and/or perceived relief from stress and boredom which are inherent in many low paid, unskilled jobs undertaken by young adults. In addition, in some social contexts smoking is used as a marker of time-out and sociability. This perhaps helps explain why the decline in young adults’ smoking is slower than that in younger adolescents where the more restrictive contexts of school and home environments are barriers to smoking uptake.[23] Our findings show how leaving these environments for more transient, independent lives can facilitate or discourage smoking. They highlight the continuing importance of maintaining smokefree policies to reduce the perceived desirable attributes of smoking. They also have wider implications in relation to the need to create more positive working environments for young adults, which include working

For peer review only - http://bmjopen.bmj.com/site/about/guidelines.xhtml

with employers, employer organisations and unions to address the culture of smoking breaks and the working hours and conditions that encourage smoking. Finally, the strong link between smoking and alcohol highlights the challenge of how to decouple further drinking from smoking, perhaps through requiring completely smokefree areas outside bars, a proposal that has found support among social smokers in New Zealand.[21] However, it also presents the opportunity of targeting socioculturally tailored smoking interventions to young adults in bars and clubs, an approach that has produced promising results in the US.[14,45,46]

Acknowledgements

The authors would like to thank all of the participants who gave their time and thoughts to the research, as well as colleagues at ScotCen Social Research; Irene Miller and Susan MacLeod for conducting the interviews, Lesley Birse for research and administrative support, and Stephen Hinchliffe and Laura Brown for data management and analysis.

Contributors

AA and AM conceived the study and designed the methods. AM oversaw participant recruitment. All authors read the transcripts and developed the analysis. HD drafted the manuscript with critical contributions from AA and AM in revised versions.

Competing Interests

The authors have no conflicts of interest to disclose.

Funding

For peer review only - http://bmjopen.bmj.com/site/about/guidelines.xhtml

This work was supported by a Cancer Research UK (CRUK) Tobacco Advisory Grant (TAG) award. Grant reference number: A21556.

Data Sharing Statement

No additional data available

For peer review only

REFERENCES

[1] Royal College of Physicians. *Nicotine without smoke: Tobacco harm reduction*. 2016. https://www.rcplondon.ac.uk/projects/outputs/nicotine-without-smoke-tobacco-harm-reduction-0 last accessed 4 October 2017.

[2] *Scottish Schools Adolescent Lifestyle and Substance Use Survey (SALSUS) 2015*. 2016. http://www.gov.scot/Topics/Research/by-topic/health-community-care/social-research/SALSUS, last accessed: 4 November 2017.

[3] European Union. *Special Eurobarometer 458- Attitudes of Europeans towards Tobacco and electronic cigarettes.* 2017. Brussels, European Union. http://ec.europa.eu/COMMFrontOffice/publicopinion/index.cfm last accessed 10 September 2018

[4] McLean J, Christie S, Gray L. *Scottish Health Survey 2016*. Edinburgh: Scottish Government, 2017. http://www.gov.scot/Publications/2017/10/2970/0 last accessed 4 November 2017.

[5] Scottish Government. *Creating a Tobacco-free Generation: A Tobacco Control Strategy for Scotland*. Edinburgh: Scottish Government, 2013. http://www.gov.scot/resource/0041/00417331.pdf last accessed 4 November 2017.

For peer review only - http://bmjopen.bmj.com/site/about/guidelines.xhtml

[6] Department of Health. *Towards a Smokefree Generation: a Tobacco ControlPplan for England.* London: Department of Health, 2017. https://www.gov.uk/government/publications/towards-a-smoke-free-generation-tobacco-control-plan-for-england last accessed 4 November 2017.

[7] Dutra LM, Glantz SA, Lisha NE, Song AV. Beyond experimentation: Five trajectories of cigarette smoking in a longitudinal sample of youth. *PLoS ONE* 2017; 12: e0171808. doi:10.1371/journal.pone.0171808.

[8] Arnett JJ. *Emerging Adulthood: The Winding Road from the Late Teens Through the Twenties*. New York: Oxford University Press, 2014.

[9] Amos A, Hastings G, Angus K, et al. *A Review of Young People and Smoking in England*. Public Health Research Consortium. 2009. http://phrc.lshtm.ac.uk/reports.html last accessed 4 November 2017.

[10] Sweeting H, Jackson C, Haw S. Changes in the socio-demographic patterning of late adolescent health risk behaviours during the 1990s: analysis of two West of Scotland cohort studies. *BMC Public Health* 2011;11:829. doi: 10.1186/1471-2458-11-829.

[11] Green MJ, Leyland AH, Sweeting H, Benzeval M. Causal effects of transitions to adult roles on early adult smoking and drinking: evidence from three cohorts. *Soc Sci Med* 2017; 187: 193-202. doi: 10.1016/j.socscimed.2017.06.018.

[12] Song A, Ling P. Social smoking among young adults: investigation of intentions and attempts to quit. *Am J Public Health* 2011;101, 1291 -1296.

[13] Kingsbury JH, Parks MJ, Amato MS, Boyle RG. Deniers and admitters: examining smoker identities in a changing tobacco landscape. *Nicotine Tob Res* 2016;18, 2130-37.

For peer review only - http://bmjopen.bmj.com/site/about/guidelines.xhtml

[14] Ling PM, Holmes LM, Jordan JW, Lisha NE, Bibbins-Domingo K. Bars, nightclubs, and cancer prevention: new approaches to reduce young adult cigarette smoking. *Am J Prev Med* 2017; 53, S78-S85.

[15] Goffman,E. The Presentation of Self in Everyday Life. Woodstock, NY: Overlook Press 1973.

[16]Fry G, Grogan S, Gough B, Conner M. Smoking in the lived world: how young people make sense of the social role cigarettes play in their lives. *Br J Soc Psy* 2008;47:763–780. doi: 10.1348/014466608X288818.

[17] Haines RJ, Poland BD, Johnson JL. Becoming a 'real' smoker: cultural capital in young women's accounts of smoking and other substance use. *Soc Health Illness* 2009;31:66–80. doi: 10.1111/j.1467-9566.2008.01119.x

[18] Wakefield M, Flay B, Nichter M, Giovino G. Role of the media in influencing trajectories of youth smoking. *Addiction* 2003;98(s1):79–103. doi:10.1046/j.1360-0443.98.s1.6.x

[19] Scheffels J. Stigma, or sort of cool: young adults' accounts of smoking and identity. *Eur J Cultural Studies* 2009;12:469-486. doi: 10.1177/1367549409342513.

[20] Nichter M, Nichter M, Carkoglu A, Lloyd-Richardson E, TERN. Smoking and drinking among college students: "It's a package deal". *Drug Alc Dep* 2010; 106, 16–20. doi: 10.1016/j.drugalcdep.2009.07.025.

[21] Hoek J, Maubach N, Stevenson R, Gendall P, Edwards R. Social smokers' management of conflicted identities. *Tob Control* 2013;22, 261-5.

[22] Côté J, Bynner JM. Changes in the transition to adulthood in the UK and Canada: the role of structure and agency in emerging adulthood. *J Youth Studies* 2008; 11: 251-268. doi: 10.1080/13676260801946464.

For peer review only

[23] Wiltshire S, Amos A, Haw S, McNeill A. Image, context and transition: smoking in mid-to-late adolescence. *J Adol* 2005; 28:603–617. doi: 10.1016/j.adolescence.2004.12.005.

[24] Marsh L, Cousins K, Connor J, Kypri K, Gray A, Hoek J. The association of smoking with drinking pattern may provide opportunities to reduce smoking among students. *Kotuitui: NZ J Soc Sci Online* 2016; 11, 72-81.

[25] Amos A, Wiltshire S, Haw S, McNeill A. Ambivalence and uncertainty: experiences of and attitudes towards addiction and smoking cessation in the mid-to-late teens. *Health Ed Res* 2006; 21, 181-191. doi: 10.1093/her/cyh054.

[26] Guillory J, Lisha N, Lee YO, Ling PM. Phantom smoking among young adult bar patrons. *Tob Control* 2017;26:153-157. doi: 10.1136/tobaccocontrol-2015-052821.

[27] Rooke C, Amos A, Highet G, Hargreaves K. Smoking spaces and practices in pubs, bars and clubs: young adults and the English smokefree legislation. *Health Place* 2013; 19:108–115. doi: 10.1016/j.healthplace.2012.10.009.

[28] Gifford H, Tautolo D, Erick S, Hoek J, Edwards R, Gray R. A qualitative analysis of Māori and Pacific smokers' views on informed choice and smoking. *BMJ Open* 2016: 6, e011415.

[29] Joosens L, Raw M. *The Tobacco Control Scale 2016 in Europe*. Association of European Cancer Societies. 2017. www.tobaccocontrolscale.org/wp-content/uploads/2017/03/TCS-2016-in-Europe-COMPLETE-LoRes.pdf last accessed 4 November 2017.

[30] Parry O, Thomson C, Fowkes FG. Life course data collection: qualitative interviewing using the Life Grid. *Soc Res Online* 1999;4. doi: 10.5153/sro.233.

[31] Bancroft A, Wiltshire S, Parry O, Amos A. 'It's like an addiction first thing … afterwards it's like a habit': daily smoking behaviour among people living in areas of deprivation. *Soc Sci Med* 2003; 56: 1261-1267.

For peer review only - http://bmjopen.bmj.com/site/about/guidelines.xhtml

[32] Ritchie D, Amos A, Martin C. Public places after smoke-free - a qualitative exploration of the changes in smoking behaviour. *Health Place* 2010; 16: 461–469. doi: 10.1016/S0277-9536(02)00124-7.

[33] Braun V, Clark, V. Using thematic analysis in psychology. *Qualitative Research in Psychology* 2006; 3 (2). Doi: 10.1191/1478088706qp063oa.

[34] McCool J, Hoek J, Edwards R, Thomson G, Gifford H. Crossing the smoking divide for young adults: Expressions of stigma and identity among smokers and non-smokers. *Nicotine Tob Res* 2012;15,552-6.

[35] Graham H. Smoking, stigma and social class. *J Social Policy* 2012; 41, 83–99.

[36] Farrimond HR, Joffe H. Pollution, peril and poverty: a British study of the stigmatization of smokers. J Comm Applied Soc Psych 2006; 16, 481–491.

[37] Leas EC, Zablocki RW, Edland SD, Al-Delaimy WK. Smokers who report smoking but do not consider themselves smokers: A phenomenon in need of further attention. *Tob Control* 2014: 24, 400-403.

[38] Gray RJ, Hoek J, Edwards R. A qualitative analysis of 'informed choice' among young adult smokers. *Tob Control* 25, 46-51.

[39] Schane RE, Glantz SA, Ling PM. Nondaily and social smoking- an increasingly prevalent pattern. *Arch Intern Med* 2009;169, 1742-1744.

[40] Jiang N, Lee YO, Ling PM. Young adult social smokers: Their co-use of tobacco and alcohol, tobacco-related attitudes, and quitting efforts. *Prev Med* 2014;69,166-171.

[41] IPPR. *Trends in Part-time and Temporary Work*. 2011. http://www.ippr.org/files/uploadedFiles/pressreleases/Part-time%20and%20temporary%20work%20technical%20briefing.pdf last accessed 5th October 2017.

For peer review only - http://bmjopen.bmj.com/site/about/guidelines.xhtml

[42] UK Parliament. *Young People in the Labour Market: Key Issues for the 2015 Parliament.* 2015.https://www.parliament.uk/business/publications/research/key-issues-parliament-2015/work/young-people-in-the-labour-market/ last accessed 5th October 2017

[43] Office for National Statistics. *UK Labour Market: September 2017*. 2017. https://www.ons.gov.uk/employmentandlabourmarket/peopleinwork/employmentandemployeetypes/bulletins/uklabourmarket/september2017#young-people-in-the-labour-market last accessed 5 October 2017.

[44] Bell R, Pavis S, Amos A, Cunningham-Burley S. Continuities and changes: teenage smoking and occupational transition. *J Adol* 1999; 22, 683-694.

[45] Fallin A, TorstenRN, Neilands B, Jordan JW, Hong JS, Ling PM. Wreaking "Havoc" on smoking: social branding to reach young adult "Partiers" in Oklahoma. *Am J Prev Med* 2015; 48, S78-S85.

[46] Kalkhoran S, Lisha NE, Torsten RN, Neilands B, Jordan JW, Ling PM. Evaluation of bar and nightclub intervention to decrease young adult amoking in New Mexico. *J Adol* 2016; 59, 222-229.

For peer review only

Timeline

Diary: a typical day

AGE	Column A<br>School/work/<br>training/unemp	Column B<br>Social context/life events	Column C<br>Smoking history (views and behaviour)<br>(For non smokers include 2nd hand experience of smoking)	Column D<br>E-Cigs history (views and behaviour)
Pre 16				
16				
17				
18				
19				
20				
21				
22				
23				
24				

For peer review only

Time	Col A<br>Activity	Col B<br>At<br>Home	Col C<br>Other<br>location	Col D<br>Smoking	Col E<br>E-Cigs	Col F<br>Reasons/influences for<br>smoking/e-cigs (or wanting to)? How feeling?
5.00 am						
5.30 am						
6.00 am						
6.30 am						
7.00 am						
7.30 am						
8.00 am						
8.30 am						
9.00 am						
9.30 am						
10.00 am						
10.30 am						
11.00 am						
11.30 am						
12 noon						
12.30 pm						
1.00 pm						
1.30 pm						
2.00 pm						
2.30 pm						
3.00 pm						
3.30 pm						
4.00 pm						
4.30 pm						
5.00 pm						
5.30 pm						
6.00 pm						
6.30 pm						
7.00 pm						
7.30 pm						
8.00 pm						
8.30 pm						
9.00 pm						
9.30 pm						
10.00 pm						
10.30 pm						
11.00 pm						
11.30 pm						
12 mid						
12.30 am						
1.00 am						
1.30 am						
2.00 am						
2.30 am						
3.00 am						
3.30 am						
4.00 am						
4.30 am						

For peer review only

Diary: Atypical Day

Time	Col A Activity	Col B At Home	Col C Other location	Col D Smoking	Col E E-Cigs	Col F Reasons/influences for smoking/e-cigs (or wanting to)? How feeling?
5.00 am						
5.30 am						
6.00 am						
6.30 am						
7.00 am						
7.30 am						
8.00 am						
8.30 am						
9.00 am						
9.30 am						
10.00 am						
10.30 am						
11.00 am						
11.30 am						
12 noon						
12.30 pm						
1.00 pm						
1.30 pm						
2.00 pm						
2.30 pm						
3.00 pm						
3.30 pm						
4.00 pm						
4.30 pm						
5.00 pm						
5.30 pm						
6.00 pm						
6.30 pm						
7.00 pm						
7.30 pm						
8.00 pm						
8.30 pm						
9.00 pm						
9.30 pm						
10.00 pm						
10.30 pm						
11.00 pm						
11.30 pm						
12 mid						
12.30 am						
1.00 am						
1.30 am						
2.00 am						
2.30 am						
3.00 am						
3.30 am						
4.00 am						
4.30 am						

For peer review only

For peer review only - http://bmjopen.bmj.com/site/about/guidelines.xhtml

Interview Guide

The interviews aim to explore:

- views of smoking, current smoking behaviour and history
- influence of peers/family/home/social networks on smoking beliefs and behaviours
- influence of social contexts – the home, local area, educational and occupational environments (and transitions)
- mechanisms for stopping smoking; what has helped smoking cessation; use of and views of NRT/e-cigarettes/vaping devices
- views, awareness and use of e-cigarettes
- (views of smoking policy/legislation)
- **For smokers**
- barriers and facilitators to quitting, and intention to quit in the future
- barriers/facilitators to interventions/support to help quit
- intention to quit in the future
- perceptions of the impact of their smoking on their own health/lives
- **For Ex-smokers**
- why and how they stopped smoking
- what helped smoking cessation: support and facilitating factors
- perceptions of any specific interventions/support they have experienced
- **For never smoked**:
- factors/issues that may be protective in preventing smoking initiation
- influence of peers and social groups, local community and social context

For peer review only

<u>Introduction/Recap</u>

To explore perceptions and experiences of smoking and quitting including views of smoking, current smoking behaviour/ history and influence of peers/family/home/social networks and context on smoking beliefs and behaviours. Study funded by Cancer Research UK.

Interview Format

- Format of depth interview (open questions, hearing their views)
- Use of grids to help completion of interview
- No right or wrong answers – their views are important/ Confidentiality
- Withdrawal at any time from interview as whole, or in not answering particular questions
- Timing of interview

Recording of Interview

- Digital recording of interviews – check they are happy with this
- Report, use of quotations, anonymization/ Check if respondent has any questions? happy to proceed?

Consent

- Obtain informed consent.

Introduction/ context (5 mins)

The aim here is to create a picture of the participant and their social context. The types of questions you could ask to get started are – Tell me a bit about yourself?

Check age and remind participant that they have been contacted as they previously took part in SHeS 2014 or 2015

Who do you live with, if anyone? Relationship with others in household/ Describe the community you live in, what is it like, how long have you lived there?

Work status and other sampling characteristics (10mins)

The aim of this section is to understand their current educational/employment status. The kind of questions to ask in this section are:

We are interested in what has been happening in your life since you were recently/around 16 years old. Can you tell us about what has been going on for you?

- Are you currently in school/college/university; employed or unemployed? **USE TIMELINE AND SHOW TO RESPONDENT TO USE AS A PROMPT.**

  Map out individual's school, work, university, training and periods of unemployment using **timeline grid (Column A)**. The following probes could be used:

  - If school/further education: what stage? What next?
  - Employed: Who with? How long for? Why change jobs? Do you like the job?
  - Unemployed? How long for? Are you receiving benefits? Any interviews, training? Any voluntary work? Youth projects?
  - Future hopes and plans

  Then map out individual's social context/life changes **(Column B)**. The following probes could be used:

  - Where were you living? Who with? Why the changes?
  - Were you renting? Own the property/pay mortgage? Parents/others own?
  - Life events: any major changes? Becoming a parent?
  - Future hopes and plans

  - ONLY IF THE INTERVIEWEES RAISE THESE ISSUES THEMSELVES PLEASE PROBE: Significant deaths of family members/ friends? Serious illnesses?
  - Problems with police, law. Note any times in young offender institutions, prison, fines, CPOs etc

Then identify **smoking status**:

- Have you ever tried smoking tobacco?
- *If never tried, go to* **<u>Never smoked section</u>**

For peer review only

Smoking initiation (5 mins)

*We are interested in your smoking history from when you first tried it to today; let's start with when you first tried it…. (Please use **Timeline Column C** for this smoking history section) Use questions such as:*

- Can you tell me all the reasons you tried smoking? (Probe fully on this) When was this? What age? Who with? How did you start? What did you think when you first tried it? Did you feel any pressure to smoke at all and who from? Did you grow up in a household where anyone smoked, and if so who did?

- What did you smoke when you first tried smoking? Tobacco, Roll-ups, Cannabis etc / who did you smoke with? Did you carry on smoking? Where did you smoke, when and how many - weekdays vs weekends? In what ways did you obtain tobacco (direct purchase, agent purchase, from friends, etc)? Which shops/friends/family members, etc?

- **If spontaneously mentions e-cigs record in Timeline Column D and probe fully.**

History for smokers – Current and ever smokers (15- 25 mins)

*This section is for those who smoke or ever smoked and we are interested in gathering a full detailed history of behaviour and exposure to smoking as well as current status. Use **Timeline Column C** for history*

Of particular interest are experiences and any changes during and after school, from initiation through the years 16 to current age. **Particularly interested in transitions** *e.g. school to work.*

*Ask in detail about **main changes** in smoking over course of history to current day -and **any factors associated with these changes**.* Here it will be important to probe on what factors cause an increase/decrease in smoking (e.g. drinking/socialising with friends; feeling anxious/stressed/unhappy; access to money; feelings of stigma, transitions etc)
Questions such as: So thinking of when you first started smoking, please take me through your smoking history. We are interested in any changes that have occurred over time.

From when you first started smoking to nowadays:

- What smoked? Type, Brand, how many – weekdays, weekends, during day vs evenings, etc – probe on reasons behind any reported changes. Cannabis smoking?

- Where usually smoke?*, ask about : different locations/nights out; home (any smoking rules?) etc*

- When did you smoke and who with? (day/ weekends /evenings)? What else were they doing when they were smoking? *Probe for smoking and drinking relationship* friends and family smoking?

- Why did you smoke? What did you enjoy and not enjoy about smoking? Any particular triggers?

- Any periods of cutting down or smoking more frequently? Why was this?

For peer review only - http://bmjopen.bmj.com/site/about/guidelines.xhtml

Quitting for Current smokers only (5-10 mins)

Quit Attempts: If attempted to quit at any time *then go through first and most recent attempt in detail (if multiple attempts) to understand process using* ***Column C (****and* ***Column D if e-cigs used)****: what prompted decision to quit, interventions aware of at the time that prompted or helped them start, get through it, what didn't help, what was offered, not offered, why went back to smoking etc. For both first and last attempt, ask questions such as (can use* ***Timeline*** *to help):*

- Why did you try to stop smoking then? How long did this attempt last? Did you manage to stop or reduce your smoking? Why did you start again?:
    - What helped/hindered the attempt? Prompts: were you aware of any leaflets, websites, ads, information? If yes, how did these help or not? School or college-based education or info?
    - Offered smoking any cessation support? Tools, aids such as nicotine gum, nicotine patches, electronic cigarette, vapes etc? Prescription, purchased or obtained in other ways? How did these help, or not?
- What is the longest period of time you have ever managed to stop smoking? What made you start again on this occasion?
- Do you want to give up smoking *now*? What, if anything, encourages you to think about quitting? What holds you back from quitting?
- Do you think you will always smoke? What if anything would make you think about stopping in the future?

Ex-Smoker 15-20 mins

Ask about first quitting attempt, if multiple, and then the successful attempt using ***Timeline Column C (****and* ***Column D*** *if e-cigs used****).*** *Probe fully on process for first and successful time, from decision to quit to starting again and anything that was different to previous attempts, and why successful attempt was different*

- When did you first try to quit? What made you <u>consider</u> quitting at this stage? Take me through this quit attempt - how did you go about it?:
    - What helped/hindered during this quit attempt? Prompts: were you aware of any leaflets, websites, ads, information? If yes, how did these help or not? School or college-based education or info?
    - Offered smoking any cessation support? Tools, aids such as nicotine gum, nicotine patches, electronic cigarette, vapes etc? Prescription, purchased or obtained in other ways? How did these help, or not? Who helped you or hindered you in quitting?
    - Why did this attempt fail, in your view? (*If only one attempt, and it was successful*):
- Successful attempt: Why was this attempt successful, in your view? Anything different in this quit attempt compared with previous ones? (PROBE on sampling characteristic too; e.g., change in employment status). What factor(s) do you think helped you *the most* to quit smoking? What factor(s) made it hardest to quit?

For peer review only - http://bmjopen.bmj.com/site/about/guidelines.xhtml

- Looking back on your successful quit attempt, is there anything you would do differently with the benefit of what you know now? Is there anything that would have made it easier for you?

- Do you think you will smoke tobacco again? Why do you say that?

Never smoked: 15 mins

*These questions are for those people who have never smoked. We are interested in understanding why they did not take up smoking when others around them did/do smoke. Can use the **Timeline** again, particularly **Column C** (but also **Column B**) as individuals may have felt pressured to smoke at different times, or may have been tempted.*

Reasons for not smoking tobacco, the type of questions you can ask here are:

- Has there ever been anytime in your life when you have been tempted to smoke? Probe fully on this. ? Were you tempted to smoke cigarettes at all? When was that and tell me a bit about that situation?
    - Who was there with you, if anyone
    - What were you doing.
    - How old were you
    - Where was it?
      What about smoking anything else? Do you or have you ever tried smoking anything else? Probe fully for details

  Have there been any times you felt pressure to try smoking? Again probe fully for who, what, age, where and why felt this pressure? What did you do instead at that time?

- What, if anything, do you think made you decide *not* to smoke tobacco? What, if anything, put you off smoking? Have these reasons for not smoking changed over time at all? If so, in what way? *Probe on factors such as personal health, cost, dislike of habit (smell etc), illness/death of family member of friend, health promotion, etc*

- Did anyone in your family smoke in the household when you were growing up? Does anyone smoke in your household now?

- Who, if anyone, do you know who smokes around you? Friends and family members? What do you think of this? Have you ever/Do you encourage(d) them to stop? Have any of them managed to quit? If so, what helped them? On the other hand, what has hindered quit attempts? How common is /was smoking in your community ?

- *Repeat questions relating to presence of smoking in their lives from pre -16 up until current age*

- Do you think smoking tobacco is something you will try at some point in the future? Why is that?

For peer review only

ASK ALL: Diary

A TYPICAL DAY (Diary)
I would like you to talk me through a typical day for you nowadays. I would like to understand in detail what you are doing from when you wake up to when you go to bed. So what you do, where you are, who you are with, what you might be feeling at that time.

Smokers- when you smoke (or use e-cigarettes) if at all

Ex-smokers- what you might be feeling at that time, when you used to smoke (or use e-cigarettes) if at all

Never smokers- are with people who are smoking or are exposed to smoking (or using e-cigarettes) if at all.

For peer review only

AN ATYPICAL DAY (Diary)
Now I would like you to talk you through a different type of day. The sort I am thinking of is a weekend day or a day when you have a different pattern/routine. Again, I would like to understand in detail what you did from when you woke up to when you went to bed. So what you did, where you were, who you were with, what you were feeling at that time

Smokers- when you were smoking (or use e-cigarettes) if at all

Ex-smokers- what you might be feeling at that time, when you used to smoke (or use e-cigarettes) if at all

Never smokers- when you were with people who were smoking (or using e-cigarettes) if at all.

We are particularly interested in what you **feel** *about smoking.*
For smokers – *habit, a certain feeling e.g. stress, anxiety, boredom,*
For non-smokers and never smokers *what they do instead of smoking when they have similar feelings to smokers.*

Views of E-cigs/vapes and use – ask all individuals (10-15mins)

*We are also interested in whether you have tried e-cigs/ vaping (***Column D on Timeline***)– (If not already covered) ask questions such as:*

- Are you aware of Ecigs i.e. cigalikes, vapes/vapourisers and box mods? Where do you get your knowledge from in relation to these different devices?
- What do you think of Ecigs, vapes and mods? What are the major pros and cons to them?
- Have you ever tried e-cigs, Vaping, Mods? If tried, do you currently use any of these? Can use **Timeline** to cover use over time
- How often did/do you use them? Do you know others who use them? If so, who? Use **Column E on Typical and Atypical days**
- *If stopped using e-cigs, vaping, mods etc go through process and reasons for stopping*

For everyone: Impact of smoking/not smoking (5 mins)

These questions relate to the perceived impact of smoking, or not smoking, on their lives, to those around them and their health. The sorts of questions you can ask here are:

- What impact, if any, do you think (not) smoking has had on you personally? Any impacts on friends or family members around you? *Probe for perceived negatives and positives on health, finances etc*

- If relevant, imagine if you had not started/started smoking all those years ago, do you think it would have made a difference to your life? If so, in what way?

For everyone: Barriers/Facilitators to smoking 10 mins

This section aims to explore the perceived barriers and facilitators to smoking and differences between age groups – under 16, and 16-24yrs
(if relevant) Questions such as the following are suggested:

More generally, we would like to ask you explore what factors may stop people smoking, either from starting smoking in the first place, or when they are active smokers.

- What do you think the main factors which stop people starting to smoke, or may reduce/stop smokers' tobacco use?
    - Is there any difference in these factors for the following age groups: under 16s, 16-19 years, 20-24 years?

- On the other hand, what do you think the main factors are which make it more likely that people will start to smoke, or may continue/increase smoking?
    - Is there any difference in these factors for the following age groups: under 16s, 16-19 years, 20-24 years?

Any other comments/questions?

- Thank respondents for their time
- Reassure re: confidentiality and ask if there is anything they would not like to be discussed/quoted in the final report
- Ask if it's okay to call back to check some of the details after the interview
- Check if participants have any questions

For peer review only

COREQ (COnsolidated criteria for REporting Qualitative research) Checklist

A checklist of items that should be included in reports of qualitative research. You must report the page number in your manuscript where you consider each of the items listed in this checklist. If you have not included this information, either revise your manuscript accordingly before submitting or note N/A.

Topic	Item No.	Guide Questions/Description	Reported on Page No.
**Domain 1: Research team and reflexivity**			
Personal characteristics			
Interviewer/facilitator	1	Which author/s conducted the interview or focus group?	20
Credentials	2	What were the researcher's credentials? E.g. PhD, MD	1
Occupation	3	What was their occupation at the time of the study?	1
Gender	4	Was the researcher male or female?	8
Experience and training	5	What experience or training did the researcher have?	1
Relationship with participants			
Relationship established	6	Was a relationship established prior to study commencement?	n/a
Participant knowledge of the interviewer	7	What did the participants know about the researcher? e.g. personal goals, reasons for doing the research	7
Interviewer characteristics	8	What characteristics were reported about the inter viewer/facilitator? e.g. Bias, assumptions, reasons and interests in the research topic	n/a
**Domain 2: Study design**			
Theoretical framework			
Methodological orientation and Theory	9	What methodological orientation was stated to underpin the study? e.g. grounded theory, discourse analysis, ethnography, phenomenology, content analysis	9
Participant selection			
Sampling	10	How were participants selected? e.g. purposive, convenience, consecutive, snowball	7
Method of approach	11	How were participants approached? e.g. face-to-face, telephone, mail, email	7
Sample size	12	How many participants were in the study?	8
Non-participation	13	How many people refused to participate or dropped out? Reasons?	n/a
Setting			
Setting of data collection	14	Where was the data collected? e.g. home, clinic, workplace	8
Presence of non-participants	15	Was anyone else present besides the participants and researchers?	n/a
Description of sample	16	What are the important characteristics of the sample? e.g. demographic data, date	7
Data collection			
Interview guide	17	Were questions, prompts, guides provided by the authors? Was it pilot tested?	8
Repeat interviews	18	Were repeat inter views carried out? If yes, how many?	n/a
Audio/visual recording	19	Did the research use audio or visual recording to collect the data?	8
Field notes	20	Were field notes made during and/or after the inter view or focus group?	n/a
Duration	21	What was the duration of the inter views or focus group?	8
Data saturation	22	Was data saturation discussed?	n/a
Transcripts returned	23	Were transcripts returned to participants for comment and/or	n/a

For peer review only

Topic	Item No.	Guide Questions/Description	Reported on Page No.
		correction?	
**Domain 3: analysis and findings**			
Data analysis			
Number of data coders	24	How many data coders coded the data?	9
Description of the coding tree	25	Did authors provide a description of the coding tree?	n/a
Derivation of themes	26	Were themes identified in advance or derived from the data?	9
Software	27	What software, if applicable, was used to manage the data?	9
Participant checking	28	Did participants provide feedback on the findings?	n/a
Reporting			
Quotations presented	29	Were participant quotations presented to illustrate the themes/findings? Was each quotation identified? e.g. participant number	9-16
Data and findings consistent	30	Was there consistency between the data presented and the findings?	9-16
Clarity of major themes	31	Were major themes clearly presented in the findings?	9-16
Clarity of minor themes	32	Is there a description of diverse cases or discussion of minor themes?	n/a

Developed from: Tong A, Sainsbury P, Craig J. Consolidated criteria for reporting qualitative research (COREQ): a 32-item checklist for interviews and focus groups. *International Journal for Quality in Health Care*. 2007. Volume 19, Number 6: pp. 349 – 357

Once you have completed this checklist, please save a copy and upload it as part of your submission. DO NOT include this checklist as part of the main manuscript document. It must be uploaded as a separate file.

For peer review only
